# Light-Assisted Catalysis and the Dynamic Nature of Surface Species in the Reverse Water Gas Shift Reaction over Cu/γ-Al_2_O_3_

**DOI:** 10.1021/acsami.4c15849

**Published:** 2024-11-29

**Authors:** Kristijan Lorber, Iztok Arčon, Matej Huš, Janez Zavašnik, Jordi Sancho-Parramon, Anže Prašnikar, Blaž Likozar, Nataša Novak Tušar, Petar Djinović

**Affiliations:** † National Institute of Chemistry, Hajdrihova 19, Ljubljana SI-1000, Slovenia; ‡ University of Nova Gorica, Vipavska 13, Nova Gorica SI-5000, Slovenia; § Jožef Stefan Institute, Jamova Cesta 39, Ljubljana SI-1000, Slovenia; ∥ Ruđer Bošković Institute, Bijenička Cesta 54, Zagreb 10000, Croatia; ⊥ Association for Technical Culture of Slovenia, Zaloška 65, Ljubljana SI-1000, Slovenia; # Institute for the Protection of Cultural Heritage, Poljanska 40, Ljubljana SI-1000, Slovenia

**Keywords:** light-assisted catalysis, reaction mechanism, in situ spectroscopy, hydroxyl, copper, RWGS

## Abstract

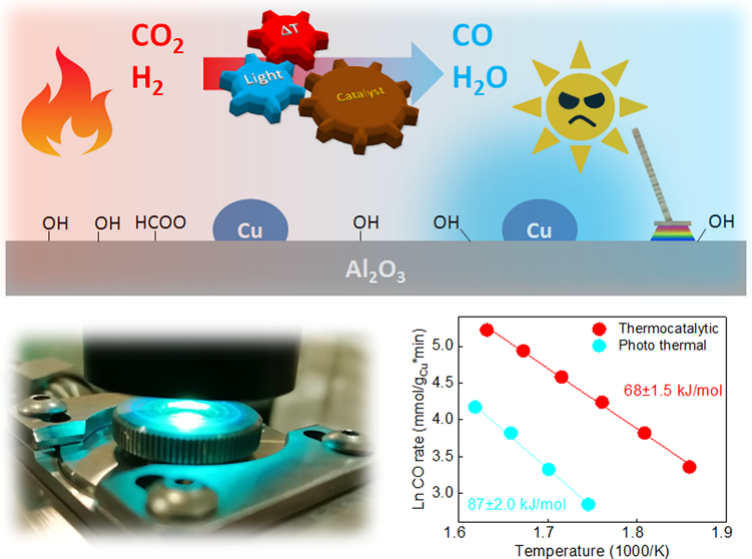

The reverse water gas shift (RWGS) reaction converts CO_2_ and H_2_ into CO and water. We investigated Cu/γ-Al_2_O_3_ catalysts in both thermally driven and light-assisted RWGS reactions using visible light. When driven by combined visible light and thermal energy, the CO_2_ conversion rates were lower than in the dark. Light-assisted reactions showed an increase in the apparent activation energy from 68 to 87 kJ/mol, indicating that light disrupts the energetically favorable pathway active in the dark. A linear correlation between irradiance and decreasing reaction rate suggests a photon-driven phenomenon. In situ diffuse reflectance infrared Fourier transform spectroscopy and TD-DFT analyses revealed that catalyst illumination causes significant, partly irreversible surface dehydroxylation, highlighting the importance of OH groups in the most favorable RWGS pathway. This study offers a novel approach to manipulate surface species and control activity in the RWGS reaction.

## Introduction

The reverse water gas shift (RWGS) reaction is an attractive approach for CO_2_ conversion to CO using renewable hydrogen^1^ and could play an important role in the transition to sustainable, large-scale CO_2_ utilization for fuel manufacturing via syngas chemistry.^2^ The RWGS reaction is a slightly endothermic equilibrium-limited reaction and is catalyzed by Cu, Ni, Pd, Pt, or Au, supported on transition-metal oxides, such as Al_2_O_3_, ZrO_2_, CeO_2_, SiO_2_, MgO, etc.^3,4^

Direct catalytic activation of CO_2_ is energetically demanding and is strongly accelerated in the presence of hydrogen. However, this can lead to a loss of CO selectivity due to competing methane formation.^3−5^ Copper-based catalysts are widely applied for RWGS reaction due to their affordability and projected large-scale applicability, good activity at low temperatures, and minimal selectivity toward methane.^6^

The metal-support interface is generally regarded as the reactive perimeter,^7^ and the RWGS reaction is reported to proceed through different pathways, such as redox, carbonate, or formate mechanisms, which are still debated.^8−12^ Recently, experimental and theoretical evidence has been mounted against the formate intermediate as an important contributor to CO synthesis.^8,13^

Light-assisted catalysis is an emerging approach toward utilizing symbiotic effects of photon energy (light) and thermal energy to accelerate the catalytic turnover and steer selectivity.^14,15^ Because of endless sunlight abundance, light-assisted or, preferably, light-driven large-scale photocatalytic reactions could play an important role in CO_2_ conversion using renewable energy. Light can assist in catalytic reactions through several mechanisms, such as interphase charge transfer, electromagnetic nearfield enhancement, or localized heating.^16^ Consequently, hot carriers (electrons and holes) can participate in the catalytic reaction via reduction at the conduction band and oxidation at the valence band or vibrational and rotational energy can be deposited into bonds of adsorbed species, resulting in their easier cleavage and formation.^17,18^ Also, light irradiation can be used as leverage to switch the oxidation state between Cu and Cu^1+^^19^ or change the distribution of adsorbed surface species, leading to altered surface chemistry.^17^ This could have important consequences for enabling more widespread use of solar irradiation to help drive catalytic reactions at mild conditions and overcome conversion and/or selectivity limitations of equilibrium reactions.^18^

Several CO_2_ hydrogenation reactions show a substantial rate acceleration in the light-assisted mode, such as CO_2_ to C_2+_ hydrocarbons,^20,21^ CO_2_ to methanol,^22,23^ methane dry reforming reaction,^24,25^ and Sabatier reaction.^26^ The light-induced acceleration of the RWGS reaction rate has been documented on Au, In-modified TiO_2_, and Fe-based catalysts.^27,28^ Interestingly, hardly any reports on photothermal RWGS studies over copper-based catalysts exist where a flow-type reactor is used, and the catalyst is excited only by a combination of thermal energy and visible light.^29^

Metallic copper is a fascinating candidate for the light-assisted RWGS reaction due to its affordability, good intrinsic catalytic activity for (thermally driven) RWGS reaction, and strong localized surface plasmonic resonance (LSPR) in the visible range of the electromagnetic spectrum. Over plasmonic metal structures (Al, Cu, Ag, Au), a resonant photoinduced collective oscillation of valence electrons can be established when the frequency of the photons matches the frequency of surface electron oscillations. This resonance produces elevated electric fields on the metal surface, which, upon decay, yields abundant and highly energetic charge carriers (hot electrons and holes).^29−32^ Alumina is among the most frequently used materials for dispersing catalytically active centers due to its chemical stability and high surface area.

In this work, we combined thermally driven and visible-light-assisted catalytic experimentation with TEM and structural analysis of Cu/γ-Al_2_O_3_ catalysts, time-resolved DFT, in situ vis, and X-ray absorption spectroscopy (XAS) and DRIFT spectroscopy to clarify the role of visible light in the RWGS reaction pathway. The CO formation rate decreases linearly with increasing illumination, and the RWGS reaction is steered through a higher energy barrier reaction channel. In parallel, irradiation with visible light can minimize the formation of methane side products. Our kinetic, structural, and diffuse reflectance infrared Fourier transform spectroscopy (DRIFTS) analyses showed that illumination causes a partly reversible CO productivity loss and a dynamic coverage with hydroxyl, formate, and carbonate species under dark and illuminated conditions.

## Results

### Catalytic RWGS Performance

**Figure 1:**
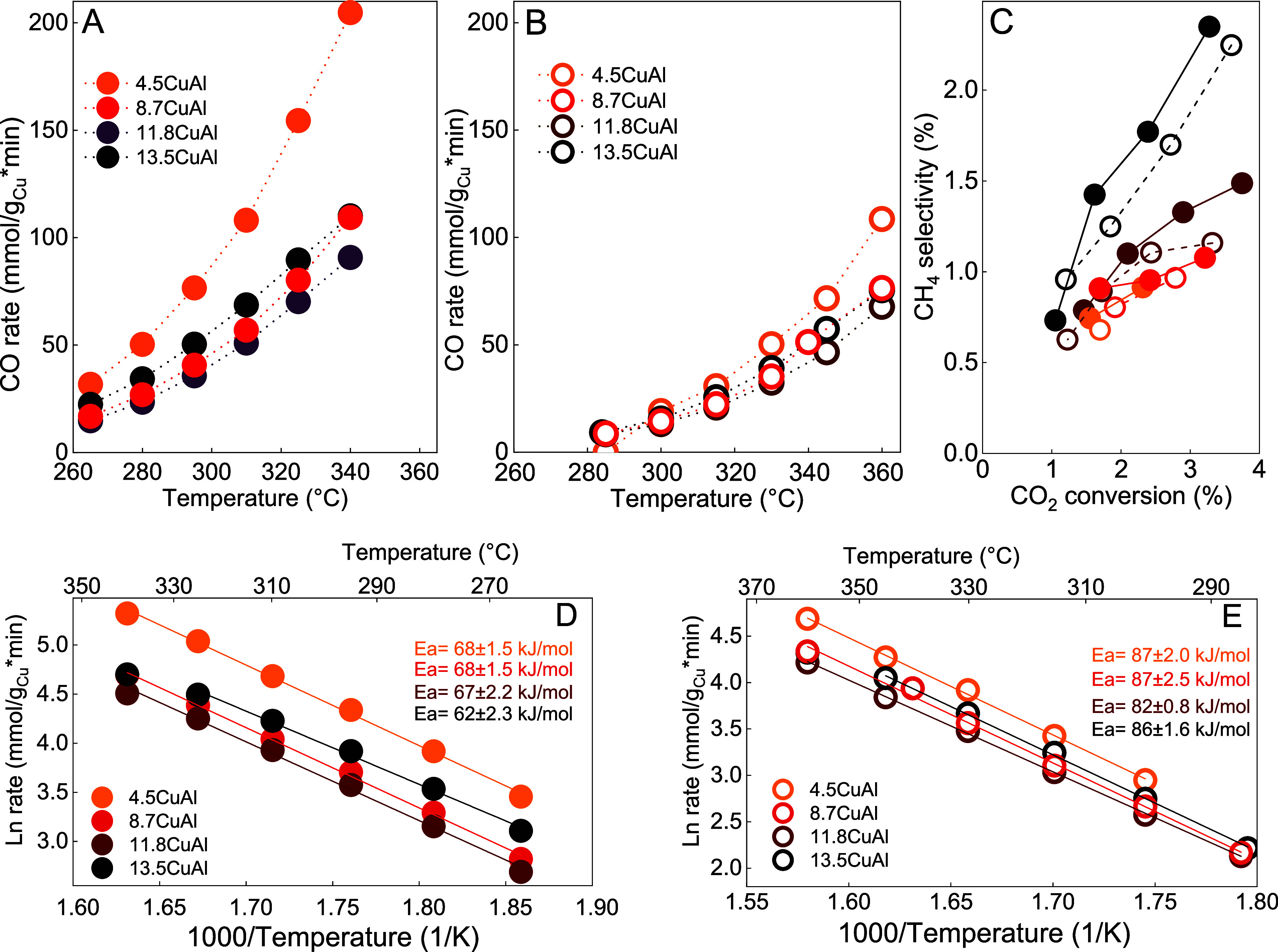
(A) Thermo-catalytic (full symbols) and (B) light-assisted (empty symbols) CO formation rates. (C) CH_4_ selectivity at identical CO_2_ conversion in thermo-catalytic (full symbols) and light-assisted modes (empty symbols); lines to guide the eye. (D) Apparent activation energies in thermo-catalytic and (E) light-assisted RWGS reaction over Cu/Al_2_O_3_ catalysts.

In the thermally driven catalytic reaction (Figure 1A), CO appears at about 260 °C, and the CO rate increases exponentially with increasing temperature, reaching 205 mmol of CO/g_Cu_*min at 340 °C. The CO rate, normalized per mass of copper, revealed the 4.5CuAl sample as the most active, whereas no distinct differences could be observed among the remaining samples with higher copper content. Similar behavior was observed previously by the group of Rodriguez over Cu–CeO_2_/ZSM-5 catalysts.^4^ Negligible deactivation of the catalyst was observed at 340 °C (Figure S1).

In the light-assisted RWGS reaction (Figure 1B), catalysts were constantly irradiated by 790 mW/cm^2^ of white light, and the catalyst temperature was varied by changing the power supplied to the resistive electric heater. The reaction was initiated at higher temperatures than in the dark, and the CO rate was consistently lower for all samples at identical catalyst temperatures compared to the thermo-catalytic experiment. The 4.5CuAl was again the most active, reaching 109 mmol of CO/g_Cu_*min at 340 °C; however, the difference in activity was much less pronounced compared to samples with higher copper content. Contrary to the commonly observed positive effect of light during light-assisted CO_2_ hydrogenation,^17,33^ visible light has a notable and negative effect on the RWGS rate over Cu/Al_2_O_3_ catalysts. Besides CO, methane was the only carbon-containing reaction product, reaching up to 2.4% selectivity (Figure 1C). Methane selectivity increased with the temperature and, more importantly, copper loading.

The role of light during the RWGS reaction was further manifested by influencing methane selectivity at identical CO_2_ conversions in thermally driven and light-assisted modes, as shown in Figure 1C. Thermally driven RWGS (full symbols) produces more methane than the light-assisted reaction (empty symbols). The difference is moderate, especially over 11.8CuAl and 13.5CuAl catalysts. For samples containing less copper, the light-induced methane selectivity modulation was minimal, indicating that illumination can be used to attenuate methane selectivity. This observation aligns with the results of Szanyi and Amal groups,^8,26^ reporting that methane is formed predominantly on extended metallic surfaces through formates as key intermediates.

Next, apparent activation energy (*E*_a_) values were compared for thermo-catalytic and light-assisted reactions, as they can provide important insight into reaction kinetics and mechanistic details. The *E*_a_ values calculated for the thermo-catalytic RWGS (Figure 1D) were 62–68 kJ/mol, whereas in the light-assisted mode (Figure 1E), the values ranged between 82 and 87 kJ/mol. The increase in the *E*_a_ values is consistent with lower RWGS rates and reveals a change in the reaction channel, which occurs through a higher energy barrier in the light-assisted RWGS mode.

**Figure 2:**
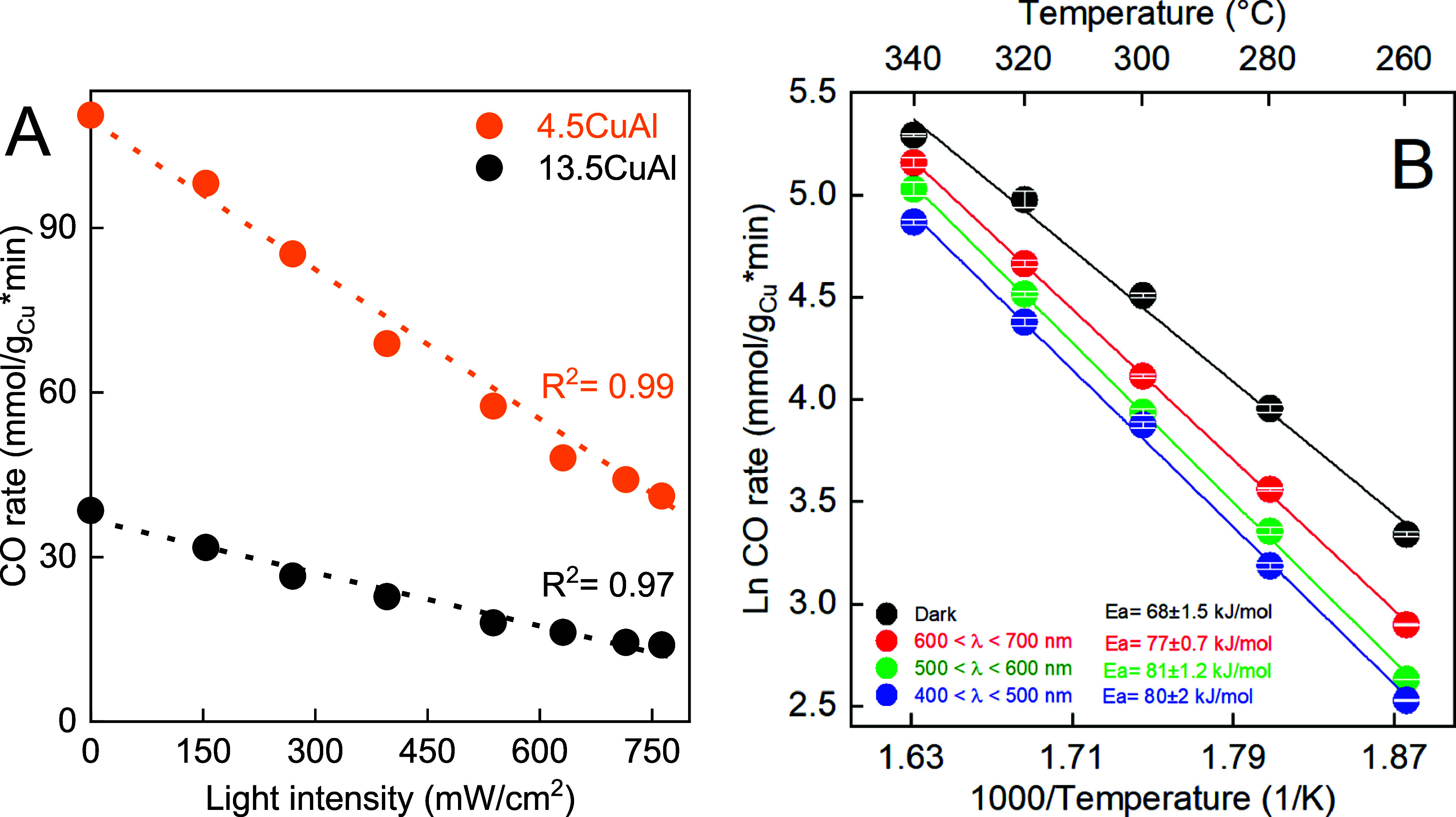
(A) Dependence of CO rates on white light intensity (400 nm > λ > 700 nm) for 4.5CuAl and 13.5CuAl catalysts at a constant catalyst temperature of 340 °C. A linear dashed line guides the eye. (B) Comparison of *E*_a_ values for 4.5CuAl catalyst in the thermo-catalytic and wavelength-dependent light-assisted RWGS reaction. Irradiance was constant at 340 mW/cm^2^.

The catalytic rate vs irradiance dependence can be used to gain mechanistic information and distinguish between thermally and photon-induced changes in reaction channels.^14^ For this purpose, white light irradiance was increased gradually from 0 to 790 mW/cm^2^ while simultaneously decreasing the power output of the electric heater, thus maintaining a constant catalyst temperature of 340 °C (Figure 2A). The experiment was performed on two catalysts containing notably different copper contents: 4.5CuAl and 13.5CuAl. In both cases, a linear negative correlation between irradiance (number of photons) and the CO rate was observed. A linear rate change suggests that the RWGS reaction is enabled (or restricted in this case) by photon absorption events, followed by interactions of the resulting hot carriers and the adsorbed species. Conversely, the heating of nanoparticles via light-induced heating shows a linear relationship between the surface temperature and irradiance, which yields an exponential dependence of reaction rate on illumination intensity.^14,34,35^

The CO formation rate over both catalysts dropped by 63–64%: from 110 to 41 mmolCO/g_Cu_*min for the 4.5CuAl, and from 39 to 14 mmolCO/g_Cu_*min for the 13.5CuAl sample.

**Figure 3:**
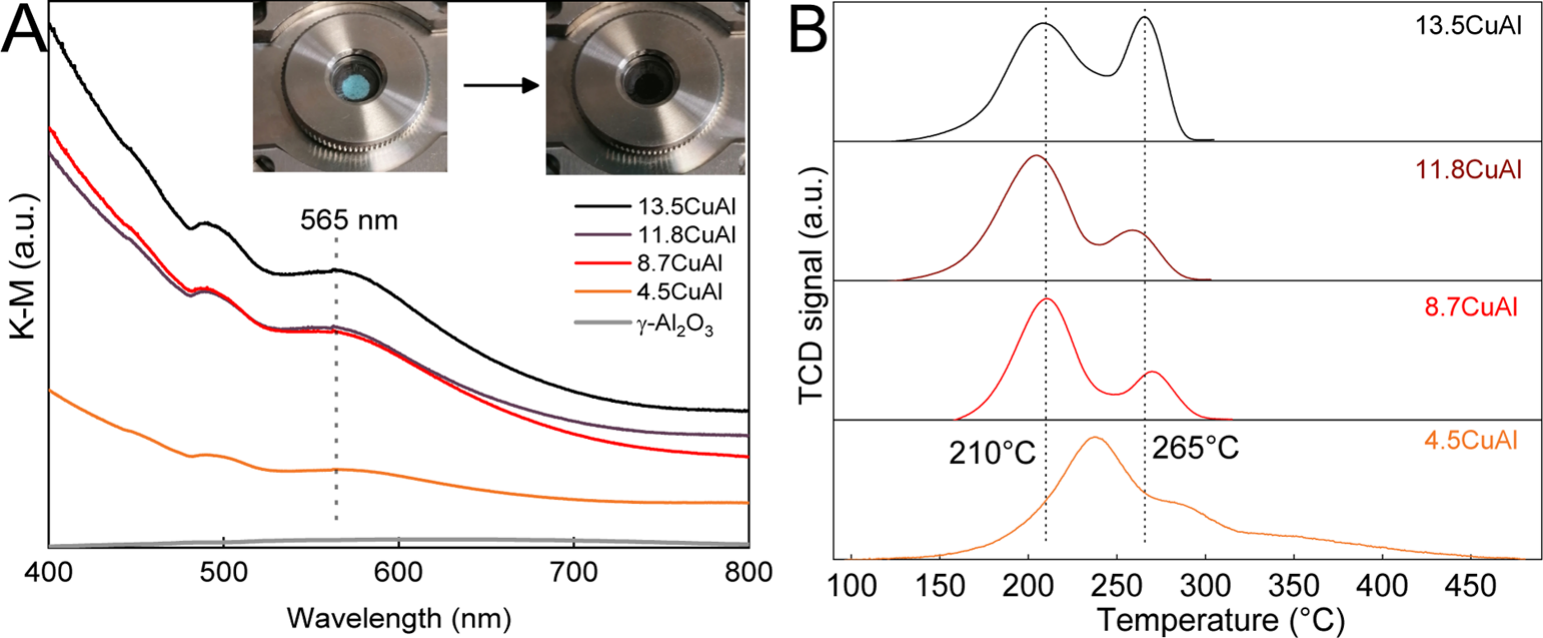
(A) In situ vis DR spectra measured at 340 °C in a 5% H_2_/N_2_ flow for the activated Cu/Al_2_O_3_ catalysts. The inset shows the color change of the 4.5CuAl sample during in situ reduction and (B) H_2_-TPR profiles of the CuAl catalysts.

Wavelength-dependent experiments (Figure 2B) were used to relate the photon absorption properties of the 4.5CuAl catalyst (Figure 3A) to modulation of the CO rate. This way it is possible to discriminate between the dominant photoexcitation processes, such as the substrate-mediated one, or direct photoexcitation of bonds formed between metal surfaces and chemisorbed adsorbates.^34,36^ For the substrate-mediated mechanism, the photocatalytic effect scales with the wavelength-dependent absorption of the catalyst, whereas direct photoexcitation of reaction intermediates occurs through electronic transitions between hybridized metal and adsorbate states, which have a completely different wavelength-dependent efficiency.

For the wavelength-dependent experiments, we used the following wavelength ranges: (i) 400–500 nm to limit excitation to inter and intraband transitions of copper,^37^ (ii) 500–600 nm corresponding to the LSPR frequency of copper,^31^ and (iii) 600–700 nm where absorption of copper is lower (Figure 3A). Irradiance was kept constant at 340 mW/cm^2^. The CO rates in all light-assisted experiments were lower than in the RWGS experiment in the dark, hence consistent with temperature-programmed catalytic data in Figure 1A,B. The absorption spectrum of the 4.5CuAl catalyst (Figure 3A) and the extent of CO rate attenuation (400–500 > 500–600 > 600–700 nm) show similar trends, suggesting the substrate-mediated mechanism is operational over the 4.5CuAl catalyst.^38,39^

Also, the *E*_a_ for CO formation over the 4.5CuAl catalyst in the dark was 68 kJ/mol (black symbols in Figure 2B). Illumination on Cu LSPR (green symbols), inter/intraband transitions (blue symbols), or with red light (red symbols in Figure 2B) increased the *E*_a_ to very similar values (77–81 kJ/mol). The hot electrons produced by inter and intraband transitions in copper have substantially lower energies than those produced by plasmon decay,^40^ yet they both attenuate the CO rate over the 4.5CuAl catalyst similarly.

### Structural and Spectroscopic Characterization

The XRD analysis of the Cu/Al_2_O_3_ catalysts (Figure S2) showed broad diffraction lines suggesting low crystallinity and/or a small crystal size, usually observed for the γ-alumina polymorph. No diffraction lines from crystalline copper-containing phases could be identified in 4.5CuAl and 8.7CuAl samples due to high dispersion and low amounts of copper. With an increase in copper amount, weak diffraction peaks emerged at 35.5, 38.7, 48.7, and 61.5° 2θ, characteristic of copper(II) oxide (monoclinic CuO). A reliable average CuO crystallite size (10 nm) could be calculated by the Scherrer equation only for the 13.5CuAl catalyst. Specific surface area and pore volume of the Cu/Al_2_O_3_ catalysts decreased gradually with increasing copper content (Figure S3 and Table S1).

Temperature-programmed reduction (H_2_-TPR) analysis was used to probe the reducible nature of copper species in the catalysts; Figure 3B. Copper-alumina interactions and copper particle size influence the redox properties of the Cu/Al_2_O_3_ catalysts. Two distinct and characteristic peaks were observed at 210 and 265 °C for the 8.7CuAl, 11.8CuAl, and 13.5CuAl samples. Reduction of smaller CuO crystals generally occurs at a lower temperature, whereas bulk-like CuO reduction occurs at higher temperatures.^41^ Increasing Cu content causes a rise in the peak at 265 °C due to a larger fraction of bulk such as Cu in these samples. The reduction profile of the 4.5CuAl catalyst was significantly different from other samples, suggesting altered morphology and chemistry of Cu species, which also correlate with the catalytic activity. The reduction was slightly delayed as the peaks shifted to 240 and 280 °C, and a shoulder was observed at about 350 °C. The shifting of reduction to higher temperatures originates from the anchoring and chemical interaction of the finely dispersed copper species with the alumina surface due to sample Cu–O–Al bond formation and an extensive copper alumina interface.^42,43^ As the copper content in the samples increases beyond 4.5 wt %, copper particles start agglomerating and growing in size, and the interaction with the support is weaker.^44,45^

The in situ vis DR spectra of the activated catalysts are shown in Figure 3A. Absorption in the entire range of wavelengths scales with increasing Cu content in the samples. A broad band with a maximum at around 565 nm originates from the LSPR of copper nanoparticles (Cu LSPR),^46^ whereas the increasing absorbance between 530 and 400 nm originates from inter and intraband electron transitions in copper.^37^ Bare γ-Al_2_O_3_ shows no interaction with visible light, consistent with its wide bandgap of 5.6 eV (Figure S4).

The effect of the copper size and shape on the absorption properties of Cu/Al_2_O_3_ catalysts was simulated (Figures S5 and S6) to validate the experimental results. Calculations closely correlate with the shape of the experimental spectra and suggest the polyhedral, near-spherical shape of copper nanoparticles. Calculations further show that the formation of Wulff facets, i.e., high truncation of the copper particles (deviation from spherical to hemispherical shape), results in more pronounced plasmon resonance and stronger electromagnetic nearfield enhancement at the metal–support interface, which should strengthen the photocatalytic effect.

**Figure 4:**
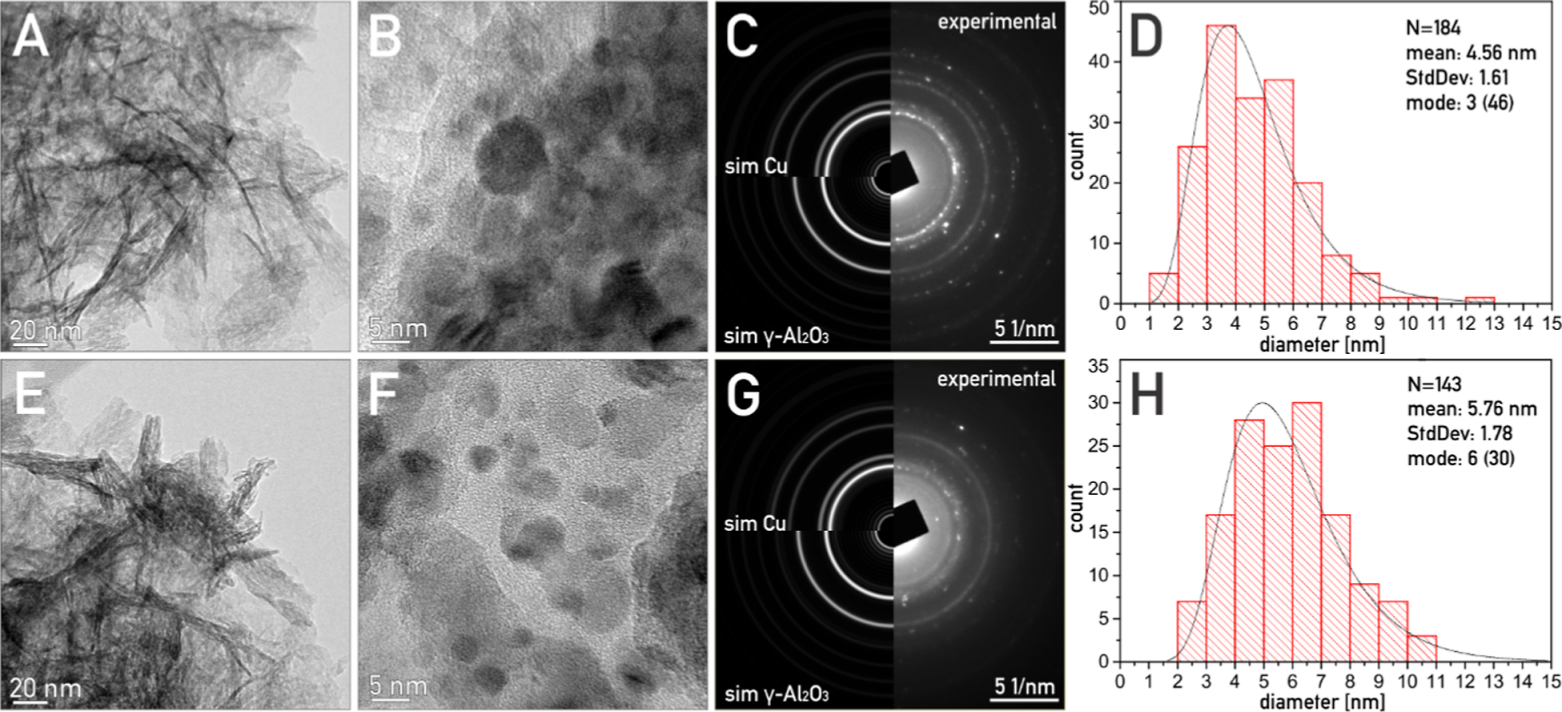
TEM micrographs, corresponding SAED patterns, and Cu particle size distribution histogram of H_2_-activated 4.5CuAl catalyst before (A–D) and after the variable irradiance experiment (E–H).

The phase composition and crystal structure of the activated 4.5CuAl catalyst (pretreated in 5% H_2_/N_2_ flow for 30 min at 340 °C) were analyzed by TEM. The γ-Al_2_O_3_ support has a flake-like morphology, with crystallites up to 5 nm in size and randomly oriented; Figure 4A,B. The γ-Al_2_O_3_ structure, as determined from selected-area electron diffraction (SAED) patterns (Figure 4C), is consistent with the XRD analysis in Figure S2.

As determined by SAED patterns, copper is present in the metallic form. The visualized Cu nanoparticles are nearly spherical, without distinct morphology and crystal facets and are evenly distributed over the alumina support. The size distribution of the visualized Cu particles varied between 1 and 13 nm, with the average size of 4.6 nm (Figure 4D).

Figure 4E–G shows the morphology of the 4.5CuAl catalyst after the variable irradiance experiment shown in Figure 2A. The alumina support shows no observable changes. Phase analysis using SAED confirmed the presence of metallic copper. However, the average copper particle size increased to 5.8 nm, which is indicative of copper sintering.

**Figure 5:**
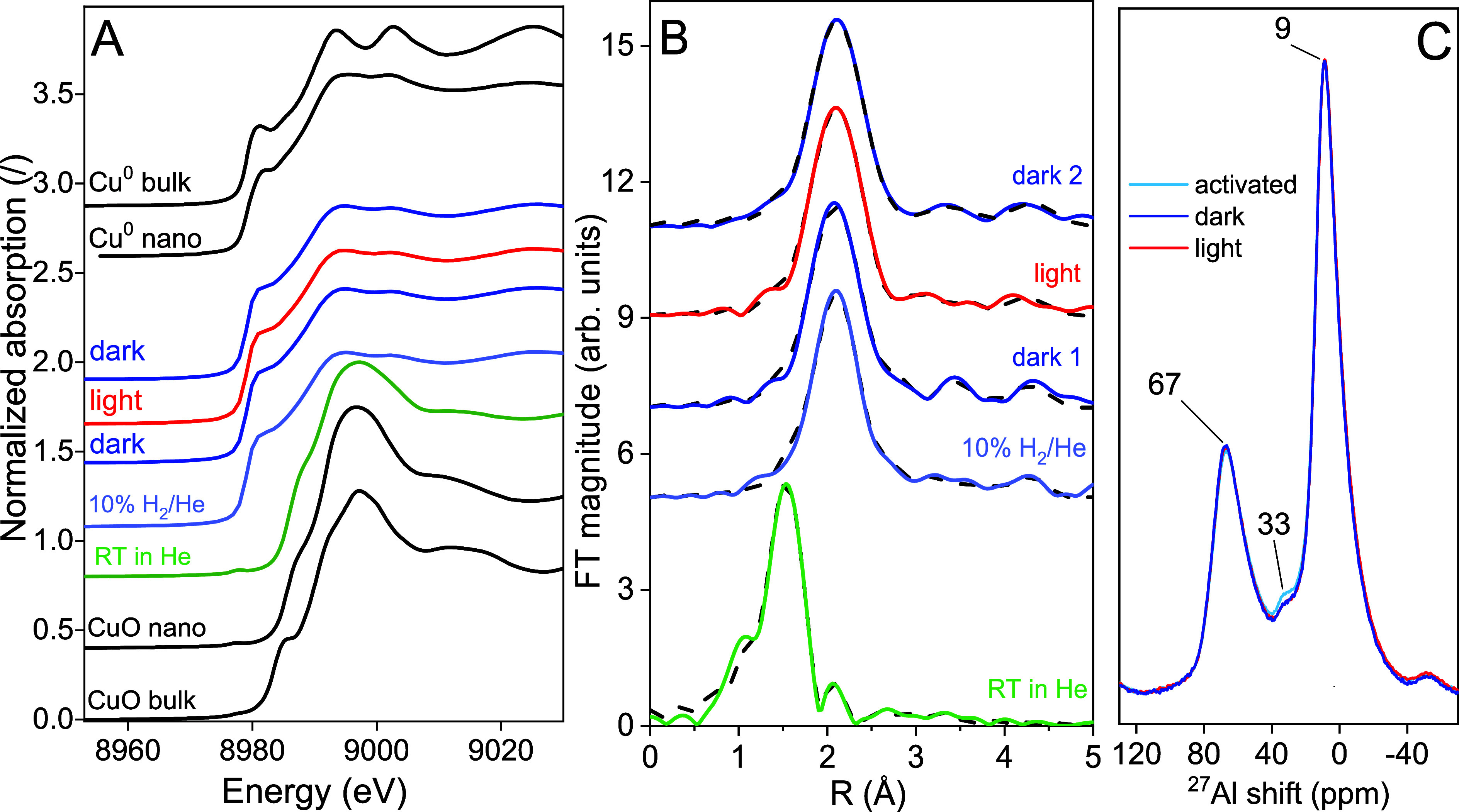
(A) Normalized Cu K-edge XANES spectra of the 4.5CuAl catalyst measured in the following sequence: RT in helium → after activation in 10% H_2_/He flow at 340 °C → thermally driven RWGS (dark 1) → light-assisted RWGS (light) → thermally driven RWGS (dark 2). The spectra of reference Cu compounds: Cu fcc metal bulk and nanoparticles; and Cu(II) oxide (CuO) bulk and nanoparticles, are plotted for comparison. (B) Fourier transform magnitude of *k*^3^-weighted Cu K-edge EXAFS spectra of the 4.5CuAl catalyst for the following sequence: RT in helium → after activation in 10% H_2_/He flow at 340 °C → thermally driven RWGS (dark 1) → light-assisted RWGS (light) → thermally driven RWGS (dark 2). Experiment (solid line) and best fit EXAFS model (dashed line). Spectra are shifted vertically for clarity. (C) Normalized ^27^Al NMR spectra of the 4.5CuAl catalyst after activation in 5% H_2_/N_2_ flow at 340 °C and after thermally driven (dark) and light-assisted (light) RWGS reaction at 340 °C.

The in situ XAS characterization using synchrotron irradiation was limited to the 4.5CuAl catalyst, which produced the largest light-induced changes in the catalytic RWGS performance. Cu K-edge X-ray absorption near edge structure (XANES) analysis was used to analyze the valence state of copper in the 4.5CuAl catalyst; Figure 5A. The energy position and shape of the absorption edge of the as-synthesized catalyst are very similar to those of reference CuO nanoparticles, indicating all copper is present as Cu^2+^ and octahedrally coordinated with oxygen atoms. The XANES spectra of the activated sample (in 5% H_2_ flow) and sample during the dark- and light-assisted RWGS reaction are all identical and exhibit energy position and edge profile characteristic exclusively for metallic Cu nanoparticles.

Quantitative analysis of in situ extended X-ray absorption fine structure (EXAFS) data (Figure 5B, Supporting Information Note and Tables S2 and S3) for the as-synthesized 4.5CuAl catalyst shows Cu^2+^ is coordinated with oxygen atoms in the first coordination shell at two different distances (on average 3.5 O atoms at 1.96 Å and 2.5 O atoms at 2.28 Å). The presence of Al neighbors at about 3 Å (on average, one Al at 2.84 Å and one at 3.04 Å) indicates that CuO clusters are attached to the Al_2_O_3_ support via Cu–O–Al bridges and that they are highly dispersed on the Al_2_O_3_ support.

In the activated 4.5CuAl catalyst, all of the Cu^2+^ is reduced to Cu^0^. The Cu–Cu neighbor distances are slightly shorter than in the Cu metal with an fcc crystal structure,^47^ and the coordination numbers of Cu neighbors are significantly lower than that in crystalline copper,^47,48^ indicating that on average, the copper nanoparticles measure below 1 nm and are highly dispersed on the alumina substrate.^47^ EXAFS analysis probes a much larger amount of catalyst than TEM, revealing that there exists a bimodal distribution of copper in the analyzed sample: most copper is present as subnanometer-sized clusters, which coexist with a small fraction of larger (4.6 nm) Cu, as visualized by TEM. A portion of Cu cations is attached to the Al_2_O_3_ support, forming Cu–O–Al bridges. On average, copper is coordinated to 1.8 O atoms at 2.71 Å, 1.9 Al atoms at 3.66 Å, and 0.9 Al atoms at 3.81 Å. The Cu–O and Cu–Al distances are significantly larger than those in the initial state before the activation (Table S2).

During catalytic reaction in the dark, the local structure of metallic Cu nanoparticles is preserved, but the coordination numbers of Al at two distances are changed notably: on average, copper is coordinated to 1.8 O atoms at 2.71 Å, 0.9 Al atoms at 3.68 Å, and 2 Al atoms at 3.81 Å, Table S2.

During the light-assisted RWGS reaction, no structural change in metallic Cu nanoparticles is detected, but the number of Al neighbors at two distances are changed: 1.8 Al atoms at 3.70 Å and 0.7 Al atoms at 3.87 Å.

When the visible-light illumination is turned off (dark 2), the Cu local structure is retained. There are no significant changes in Cu metallic nanoparticles or the Cu–O–Al bridges.

To summarize, the in situ XAS data reveal that copper remains fully metallic during the thermally driven and light-assisted RWGS reaction. Illumination with visible light causes permanent structural changes in the coordination of Cu metallic nanoparticles to the Al_2_O_3_ support (change of Cu–O–Al bridges), which also persist after the illumination is turned off.

Additional information regarding the restructuring of alumina in the 4.5CuAl catalyst was obtained by ^27^Al MAS NMR analysis; Figure 5C. Aluminum is present in three different coordination environments, namely Al^IV^, Al^V^, and Al^VI^, with chemical shifts of 67, 33, and 9 ppm, respectively. Asymmetric line shapes with a tail on the low-frequency side result from a distribution of quadrupolar coupling constants indicative of disorder in the analyzed samples. The presence of penta-coordinated Al^V^ is a direct consequence of structural disorder and relatively low crystallinity of the alumina. The presence of Al^V^ signal is slightly diminished after the dark RWGS reaction, and no further changes are observable after the light-assisted RWGS. This results from Al ion migration from penta-coordinated Al^V^ sites to the more stable hexa-coordinated Al^IV^ and tetra-coordinated Al^VI^ sites under reaction conditions. This stabilizes the alumina structure, which is likely accompanied by local crystallization. The observed changes are minor and appear to be related to only the surface of the catalyst.

**Figure 6:**
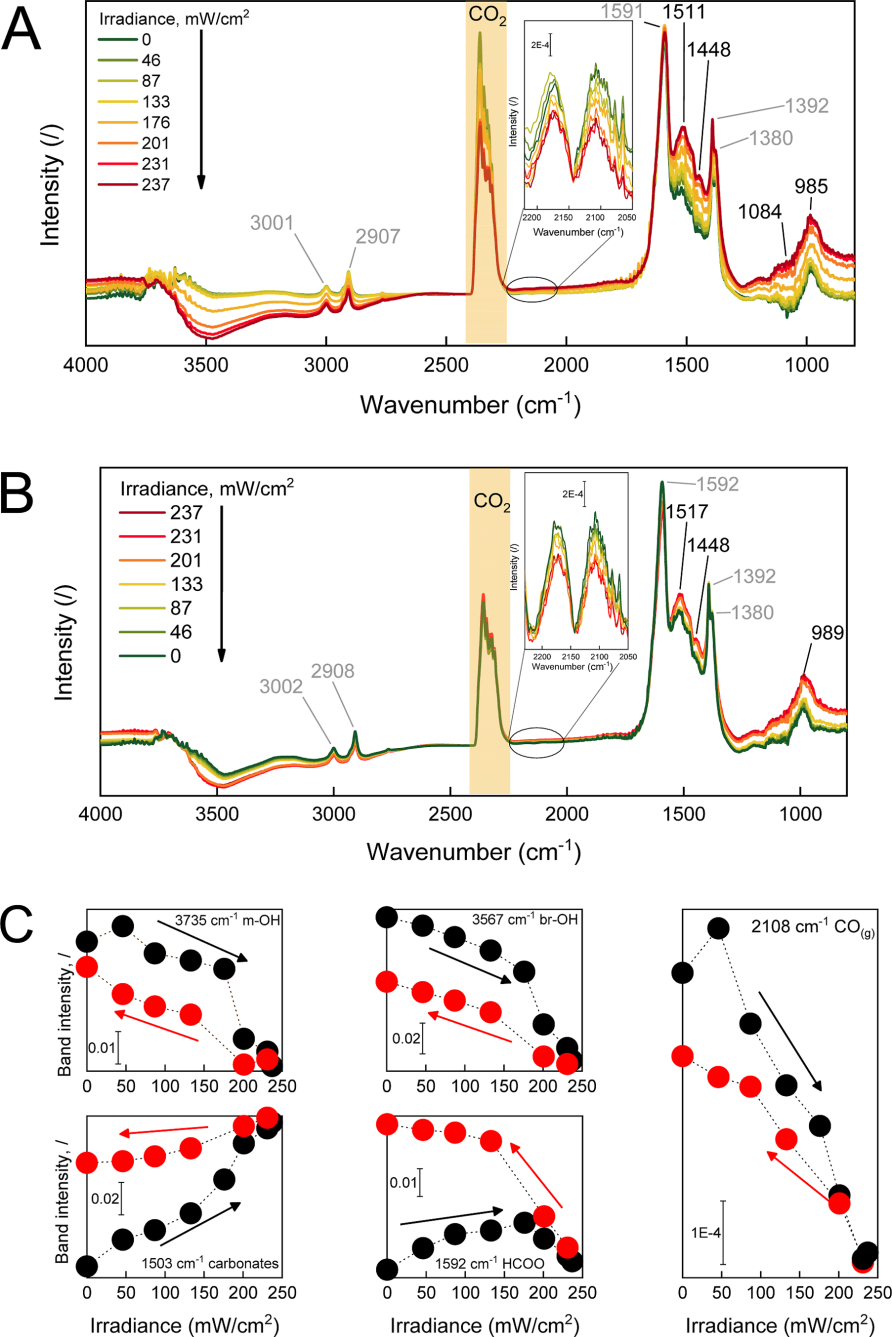
(A) In situ DRIFT spectra during RWGS reaction over the 4.5Cu catalyst at a constant temperature of 340 °C and gradually increasing irradiance (0 → 237 mW/cm^2^) and (B) same catalyst during gradually decreasing irradiance (237 → 0 mW/cm^2^). The inset shows the characteristic CO_(g)_ bands, confirming light-induced drop of catalytic activity. (C) Changes of characteristic band intensity during stepwise dark → light (0 → 237 mW/cm^2^, black symbols) and light → dark transition (237 → 0 mW/cm^2^, red symbols).

We further analyzed the dynamics of surface species during the RWGS reaction at a constant catalyst temperature over a dark and illuminated 4.5CuAl catalyst by in situ DRIFT spectroscopy; Figure 6.

First, we probed and ruled out the existence of the redox RWGS mechanism, which involves copper oxidation state switching in the following manner: Cu^0^ + CO_2_ → CuO + CO, followed by CuO + H_2_ → Cu^0^ + H_2_O. Our pulse experiments over the reduced 4.5CuAl catalyst show no CO_2_ dissociation and CO formation in the dark and under illumination at 340 °C (see Supporting Information Text and Figure S7).

Initially in the dark (green line in Figure 6A), the surface species consist of asymmetric and symmetric υ(O–C–O) and υ(CH) vibrations. Formates give rise to bands at 1591 cm^–1^ υ(O–C–O)_asym-br_, 1392 cm^–1^ δ(CH), 1380 cm^–1^ υ(O–C–O)_asym-br_, and υ(CH)_sym,asym_ 2907, 3001 cm^–1^.^49,50^ Several carbonate υ(O–C–O)_sym-asym_ vibrations are located between 1400 and 1550 cm^–1^ and υ(CO) stretching between 950 and 1100 cm^–1^ reveal the presence of bidentate and monodentate carbonate species.^49,51−53^

Progressively increasing the irradiance and maintaining the catalyst temperature constant at 340 °C (Figures 6A and S9) causes dehydroxylation of the surface, as can be inferred from the decreasing signal intensity between 2700 and 3800 cm^–1^. The wide range of hydroxyl bands is due to the large variety of surface sites (Al^IV^, Al^V^, and Al^VI^), which can participate in different combinations of multibonding hydroxyls.^54−56^ In addition, the carbonate O–C–O bands between 1400 and 1550 cm^–1^ notably gain intensity, as well as the C–O bands between 950 and 1100 cm^–1^. The gas-phase CO signal (2100–2220 cm^–1^; Figure 6A inset and Figure 6C) decreases with increasing irradiance, which is consistent with ∼25% lower catalytic activity in light-assisted mode (irradiated by 250 mW/cm^2^; Figure 2A), compared to the thermally driven catalytic mode. Gradually increasing irradiance of the catalyst under isothermal conditions causes monodentate (m-OH) decomposition and bridging hydroxyls (br-OH) from the alumina surface and their substitution with carbonates, as shown in Figure 6C.

Figure 6B shows the evolution of surface species during a stepwise transition from an illuminated sample (red trace) back to the dark (green trace). Most notably, only partial rehydroxylation of the surface occurs. The monodentate hydroxyl band at 3735 cm^–1^ almost reaches its initial intensity, whereas the recuperation of the 3567 cm^–1^ band, which belongs to bridging hydroxyl groups, recovers only about 50% of the initial intensity (Figure 6C). Also, a decrease of carbonate bands, O–C–O and C–O, occurs, together with an increase of the CO gas signal (inset Figure 6B, 2100–2220 cm^–1^ and Figure 6C), revealing partial regeneration of catalytic activity. The initial CO productivity was after the isothermal dark → light → dark spectroscopic experiment lowered by 30%, showing illumination causes irreversible deactivation of the catalyst. From the difference IR spectrum (Figure S8), we can also observe the intensity increase of the characteristic formate bands, 1595 cm^–1^ υ(O–C–O)_asym-br_, 1391 cm^–1^ δ(CH), 1378 cm^–1^ υ(O–C–O)_asym-br_, and υ(CH)_sym,asym_ 2908 and 3002 cm^–1^.

The effect of temperature on the surface species distribution was also analyzed in thermally driven mode and light-assisted mode by varying the catalyst temperature between 250 and 340 °C; Figure S9. The intensity of the CO signal shows an exponential increase as a function of temperature, following the expected Arrhenius-type temperature dependence of catalytic reactions. The trends of signal intensities with increasing temperature differ compared to the isothermal ones in Figure 6C. As a result, we can conclude that (a) light and thermal energy influence the surface population and catalytic activity in very different ways and (b) the localized sample heating due to illumination can be ruled out as the origin of the observed spectral changes.

**Figure 7:**
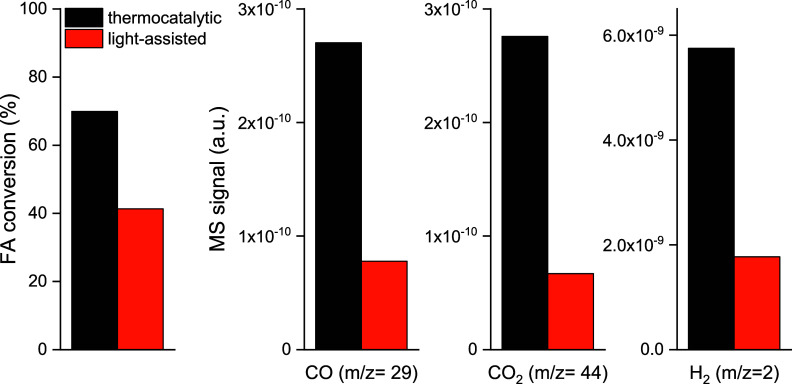
FA conversion to CO_2_, H_2_, and CO under thermo-catalytic and light-assisted modes over the 4.5CuAl catalyst at 340 °C.

The formate decomposition pathway was analyzed further to determine the light effect on this possible reaction channel, as shown in Figure 7. Formic acid (FA) adsorbs on alumina surface via deprotonation of the carboxylic group, resulting in the surface population with formate and H species.^57^ The decomposition pathway can be either dehydrogenation (producing H_2_ and CO_2_) or dehydration (producing H_2_O and CO).^58^ Dehydration is the desired reaction channel in the RWGS reaction. FA conversion was about two times higher in the thermo-catalytic mode, revealing higher reactivity of formate in the dark. However, the formate decomposition pathways under dark- and light-assisted conditions are very similar, producing both CO and CO_2_ in very similar ratios. To summarize, light decreases the reactivity of surface formate species but does not influence the products of its decomposition.

### Time-Dependent DFT Analysis of Al_2_O_3_ Surface (De)hydroxylation

Next, we applied ab initio TD-DFT calculations to investigate water adsorption on alumina, its dissociation, and consequent alumina (de)hydroxylation in the ground and (illuminated) excited states. Pristine (stoichiometric) γ-Al_2_O_3_ is reactive due to unsaturated (Lewis) Al sites and readily binds water to get hydroxylated. A water molecule binds strongly (*E*_ads_ = −1.54 eV) to the exposed aluminum atom with an Al–O distance of 1.97 Å. Upon overcoming a very low barrier of 0.28 eV, H_2_O dissociates to H, which binds to a surface oxygen atom, and OH, which binds to two Al atoms. The dissociation itself (Δ*E* = −2.16 eV) and the overall process of water adsorption and surface hydroxylation (Δ*E* = −3.67 eV) are strongly exothermic. Subsequent adsorption of water molecules is also favorable with energies of −1.55, −1.38, and −0.64 eV and dissociation energies of −0.33, −0.57 and −0.38 eV, respectively.

**Figure 8:**
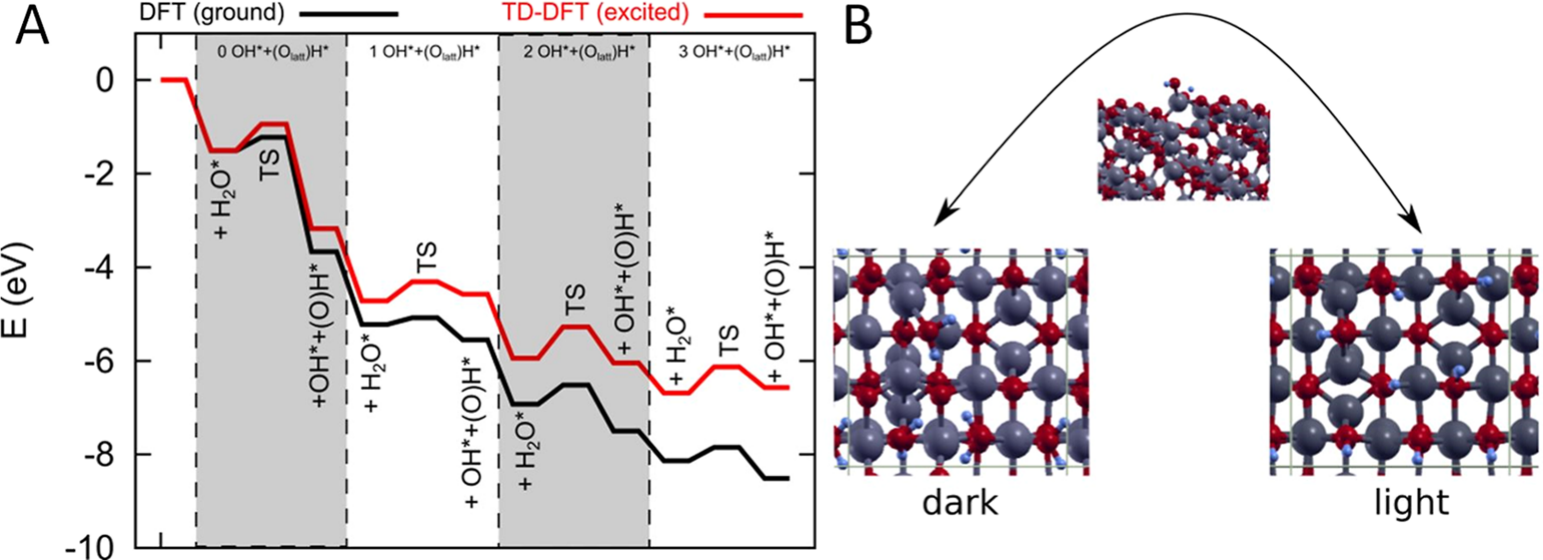
(A) Energy diagram for the adsorption and dissociation of H_2_O to OH* and (O_latt_)H* on [100] γ-Al_2_O_3_ in the dark and under illumination. (B) Geometric structures of (left) a fully hydrated and (b) fully hydroxylated [100] γ-Al_2_O_3_ surface, depending on the illumination. A transition state (top) for dissociating a single H_2_O molecule is shown.

Experimentally, we observed considerable dehydroxylation of the surface under illumination. Figure 8 shows the energy profile for water adsorption and dissociation into OH* and H* (bound to lattice O, denoted as (O_latt_)H*) on the [100] surface of γ-Al_2_O_3_. Per unit cell, a maximum of four H_2_O molecules can dissociate and hydroxylate the surface. We observe that the overall reaction of a water molecule adsorption and dissociation becomes *less* exothermic by 0.49 eV. Consequently, the barrier in the reverse direction is *decreased* by 0.21 eV (0.49–0.28 eV). This confirms that surface hydroxylation is less stable upon irradiation than under dark conditions.

Moreover, the barrier for H_2_O dissociation after adsorption is increased by 0.28 eV. Subsequent hydroxylation becomes less unfavorable. While the adsorption energies of water molecules remain relatively unchanged (−1.55, −1.37, and −0.65 eV), the dissociation energies are +0.122, −0.13, and +0.10 eV. This hints at a much more difficult surface hydroxylation under illuminated conditions.

## Discussion

Based on the catalytic, computational, and spectroscopic data, we identified the following differences in the occurring reaction pathways during the thermo-catalytic and light-assisted RWGS reactions. Visible-light illumination of the Cu/Al_2_O_3_ catalyst negatively affects the CO formation rate. The rate-slowing scales linearly with the number of photons, revealing that it is a photon-driven event that causes deactivation, not a temperature-induced phenomenon. Using TD-DFT, we have shown that in the excited state (light conditions) OH groups on the alumina surface are less stable than in the ground state (dark conditions), which is consistent with substantial dehydroxylation upon illumination. The continuous copper surfaces favor methane production, as their selectivity scales with increasing copper content and particle size. Irradiation with visible light induces a surface plasmon resonance effect on copper and localized near field enhancement, which causes dehydroxylation of the copper-alumina interface, while simultaneously increasing the carbonate population. Hydroxyl groups appear to be important in enabling low-energy barrier CO production.

EXAFS analysis revealed irreversible changes in the binding of copper cluster to the alumina support through a notably higher number of Cu–O–Al bonds. These changes in the metal–support interface are induced by extensive light-induced dehydroxylation. Copper is present as fully metallic subnanometer nanoparticles, which remain structurally intact during the reaction. The group of Pavanello^59^ analyzed γ-Al_2_O_3_ surface restructuring as a result of dehydroxylation using DFT and observed that removal of the terminal and bridging hydroxyls situated in the interstitial space, bridged between two octahedral Al atoms, resulted in substantial surface distortions, which included surface Al atoms moving toward the vacuum. Also, the bond lengths of surface Al–O atoms also become shorter, which correlates with our experimentally observed shortening of the average Cu–Al distance from 3.81 to 3.63 Å (Table S2). The Cu–Al_2_O_3_ interface and thus most active sites are different under dark and illuminated conditions.^60^ Also, the *E*_a_ for CO formation increased from 68 kJ/mol in the dark to 82 kJ/mol under illumination, revealing that the reaction mechanism is altered in light-assisted mode. This suggests an important role of interface hydroxyls in enabling the low-energy-barrier RWGS pathway, which is likely involved in the rate-determining step of the RWGS reaction.^61^ The surface hydroxyl groups and metal-support perimeter are important for the conversion of carbonate to carboxylate and its further decomposition to CO and water. Both groups of Szanyi and Mavrikakis ascribed great importance to the hydroxyl for enabling the formation of carboxyl intermediate at the Cu–O–Al interfacial sites, which was both theoretically and experimentally proven as the least energy-intensive reaction pathway during RWGS.^8,62^ Accumulation of carbonates on the catalyst surface in our study suggests that their low reactivity represents one of the most significant kinetic barriers in the CO-forming reaction. The steps involving COOH decomposition with an OH adsorbate have lower activation energies on Pt and Cu compared to decomposition with a free site, further confirming the relevance of hydroxyls in the lowest energy barrier RWGS mechanism.^62^ An additional indicator that the carboxyl-mediated mechanism is also dominant over Cu/Al_2_O_3_ catalysts is the similarity between *E*_a_ values over different Pd-based catalysts calculated by Szanyi et al. (72 ± 7 kJ/mol)^8^ and the ones in this work (68 kJ/mol). The same authors state that *E*_a_ is around 110 kJ/mol for the formate pathway. With the correlation between the lower CO rate during the RWGS reaction and the attenuated formate reactivity during illumination (Figure 7), we can conclude that the formate pathway has an contributing role to the CO formation.^63^

## Conclusions

Visible-light illumination of the Cu/Al_2_O_3_ catalyst negatively affects the CO formation rate, which is ascribed to a lower coverage with OH groups. The rate-slowing scales linearly with the number of photons and is wavelength dependent, revealing that it is a photon-driven event that causes deactivation and not a temperature-induced phenomenon. Using TD-DFT, we have shown that in the excited state (light conditions), OH groups on the alumina surface are less stable than in the ground state (dark conditions), which is consistent with substantial dehydroxylation upon illumination. The continuous copper surfaces favor methane production, as its selectivity scales with increasing copper content and particle size. Irradiation with visible-light induces a surface plasmon resonance effect on copper and localized near-field enhancement, which causes dehydroxylation of the copper–alumina interface while simultaneously increasing the carbonate population. Hydroxyl groups appear to be important in enabling low-energy barrier CO production, likely through the carboxylate intermediate. Hydroxyl abundance plays a crucial role in various catalytic reactions, such as CO_2_ hydrogenation, Fischer–Tropsch synthesis, selective catalytic reduction of NO, and Lewis acid-catalyzed dehydration.

## Methods

High-purity γ-Al_2_O_3_ (Silkem d.o.o., Slovenia) was used to support copper nanoparticles (5–15 wt % Cu). Different amounts of Cu(NO_3_)_2_·*x*3H_2_O were dissolved in 20 mL of ultrapure water under stirring. Then, 1 g of γ-Al_2_O_3_ was added. After 30 min of stirring on a magnetic stirrer, a 2.5 wt % aqueous NH_4_OH solution was added dropwise to increase the pH value to 8. The suspension was left stirring for 30 min. Afterward, pH was increased to 10 using a 25 wt % aqueous NH_4_OH solution, and the mixture was left stirring for an additional 2 h. After filtration, the catalyst precursor was dried overnight in a laboratory drier at 70 °C and calcined in a chamber furnace for 4 h at 350 °C in static air. Inductively coupled plasma optical emission spectroscopy (ICP OES) analysis revealed 4.5, 8.7, 11.8, and 13.5 wt % of copper in the synthesized samples. In the article, catalysts are denoted as xCuAl, where x represents the copper content.

XRD analyses were performed on a PANalytical X’pert PRO diffractometer using Cu Kα radiation (λ = 1.5406 Å). The analyzed 2θ range was between 20 and 80°, with a step size of 0.034° and a measuring time of 1000 s at each step.

The BET-specific surface area, Barrett-Joyner-Halenda (BJH) total pore volume, pore size distribution, and the average pore size were determined from N_2_ adsorption/desorption isotherms measured at 77 K (Micromeritics, model TriStar II 3020). The samples were degassed by using a SmartPrep degasser (Micromeritics) in a N_2_ stream for 16 h at 300 °C.

The size distribution and chemical state of copper were analyzed by TEM (JEM-2100, JEOL Inc.), operating at 200 kV and equipped with a slow-scan CCD camera (Orius SC-1000, Gatan Inc.). The powdered samples were dispersed in ethanol, sonicated for 30 s in an ultrasonic bath to prevent agglomeration, and then transferred onto commercial amorphous lacey carbon Ni-supported grids. The SAED patterns were simulated by JEMS electron microscopy simulation software, V4.9.

H_2_-TPR was performed using 100 mg of sample in a 25 mL/min flow of 5% H_2_/Ar (Micromeritics Autochem 2920). Before analysis, the catalyst was pretreated in situ in a 10% O_2_/He flow by heating the sample to 300 °C with a rate of 20 °C/min, followed by an isothermal step of 20 min. To remove water and prevent its interference with the TCD signal, an LN_2_-isopropanol cold bath was employed.

Solid-state magic-angle spinning (MAS) NMR spectra were recorded on a 600 MHz Varian NMR system equipped with a 1.6 mm HXY CPMAS probe. Before NMR measurements, samples were packed in a rotor and dried at 100 °C under a vacuum for 2 h. The sample rotation frequency was 20 kHz, while the Larmor frequency for ^27^Al was 156.16 MHz. A 0.6 μs excitation pulse was used; 2500 scans were collected, and the delay between the scans was 0.2 s. The frequency axis of ^27^Al was referenced to a 0.1 M Al(NO_3_)_3_ aqueous solution. The spectra were processed by using ssNake software.

In situ vis DR spectroscopy was performed on a LAMBDA 650 spectrophotometer (PerkinElmer) and the reaction chamber from Harrick. Spectralon was used to record the background. Finely powdered samples (2 mg) were activated in a 5% H_2_/Ar flow at 340 °C for 30 min. The simulated RWGS feed comprised 61% H_2_, 26% CO_2_, and 13% N_2_ (50 mL/min) at 340 °C.

In situ DRIFTS analysis was performed using a Frontier spectrometer (PerkinElmer) equipped with an LN_2_ MCT detector. Spectra were collected between 800 and 4000 cm^–1^, averaged over 64 accumulations with a spectral resolution of 4 cm^–1^. The powdered sample (2 mg) was placed inside the Harrick reaction chamber. The catalyst (2 mg) was pretreated in a 5% H_2_/N_2_ flow for 30 min at 340 °C, followed by switching to 61% H_2_, 26% CO_2_, and 13% N_2_ flow (50 mL/min). The sample was illuminated with white light using the Schott KL2500 LED source.

In situ Cu K-edge XANES and EXAFS experiments were performed in transmission detection mode at the BM23 beamline of the ESRF synchrotron radiation facility in Grenoble, France. XAS spectra were measured on the 4.5CuAl catalyst at RT in helium flow, after activation (10% H_2_/He flow, 30 mL/min at 1 bar and 340 °C), and during the catalytic reaction in CO_2_/H_2_/He stream (flow rate 7 mL/min of H_2_, 3 mL/min of CO_2_ and 20 mL/min of He) at 1 bar at 340 °C, with and without visible-light illumination of the catalyst. See Supporting Information for additional experimental details and results (Figure S10 and Tables S2 and S3).

The formate decomposition reaction was performed using 5 mg of the 4.5CuAl catalyst powder positioned over the SiC layer, forming a 0.5 mm thick catalytic layer with a 4.5 mm diameter. The sample was reduced at 340 °C in 25 mL/min of 5% H_2_/N_2_ flow for 30 min. Afterward, flow was changed to argon (30 mL/min), and the sample was degassed at 340 °C until all analyzed MS traces reached steady state (about 2 h). To expose the catalyst to the FA vapors, argon (30 mL/min) was bubbled through a saturator containing FA (thermostated at 23 °C) and passed through the catalyst bed. The experiment was performed in the dark and in light-assisted mode by illuminating the catalyst with 790 mW/cm^2^ of white light. Mass spectroscopy was used to simultaneously analyze H_2_ (*m*/*z* = 2), H_2_O (*m*/*z* = 18), CO (*m*/*z* = 29), CO_2_ (*m*/*z* = 44), argon (*m*/*z* = 40), and FA (*m*/*z* = 46) at the reactor outlet. The FA conversion was calculated directly from the *m*/*z* = 46 intensity, whereas the formation of H_2_, CO, and CO_2_ was estimated from their corresponding signals and compensated for the signal overlap (i.e., contribution of FA to the 44, 29, and 18 signals). Water signal is not shown because condensation on the reactor walls prevented accurate signal quantification.

The electrodynamic properties (extinction, scattering, and near field distribution) of Cu nanoparticles supported on the γ-Al_2_O_3_ substrate were investigated via numerical simulations. Effects of particle shape (truncation), size, and possible presence of Cu_2_O at the particle–substrate interface are analyzed. Simulations are performed using the boundary element method as implemented in the MNPBEM toolbox.^64^ Optical constants of Cu^65^ and Cu_2_O^66^ are taken from the literature, while γ-Al_2_O_3_ is considered transparent and has a refractive index equal to 1.72 in the considered spectral range. It is assumed that air surrounds particles and the substrate.

For quantum chemical calculations, a plane-wave formalism of the density functional theory was used as implemented in VASP 6.3.1.^67^ To keep the computational cost manageable, we employed the PBE functional and the projector-augmented-wave approach.^68^ D3 corrections by Grimme were included.^69^ An energy cutoff of 500 eV sufficed for well-converged results.

The γ-alumina surface was studied in the excited state using TD-DFT simulations implemented in VASP to provide insights into surface hydroxylation during dark/illumination.^70^ A gamma-centered one-point (Γ) mesh was used for sampling the Brillouin zone due to the computational cost. For consistency, the same mesh was used in conventional GGA DFT calculations. A bulk γ-Al_2_O_3_ structure by Digne et al.^71^ was first optimized to arrive at a unit cell of 5.55 Å × 8.36 Å × 8.03 Å, which is consistent with the experimentally determined values 5.59 Å × 8.41 × Å 8.07 Å for the *P*2_1_/*m* space group of γ-Al_2_O_3_. A slightly underestimated cell volume is due to the inclusion of D3 dispersion correction, which is known to underestimate lattice parameters but provides superior interaction energies.

Since the atomic lattice of the crystallographic model by Digne et al.^71^ is rotated by 45° relative to a conventional face-centered cubic, its [100] surface in the unit cell model corresponds to the conventional [001] surface, which is the most stable. Our investigation focused on the [100] γ-Al_2_O_3_ surface with a supercell of 8.36 × 8.04 Å, containing 6 layers of atoms (12 unit cells, resulting in Al_24_O_36_).

Catalytic tests were performed in a reaction chamber (Harrick), which is described in our previous work.^18^ For all tests, 2 mg of powdered catalyst was used, which formed a round pellet with a 4.5 mm diameter and 0.5 mm thickness. The catalyst was positioned on a 1 mm thick layer of powdered SiC (SiCat, 30–150 μm) to improve heat transfer from the furnace to the sample and minimize the radial temperature gradient in the catalyst layer. The catalyst temperature was measured with a 0.25 mm K-type thermocouple located 0.3 mm below the surface of the catalytic layer. See Figure S11 for reactor setup details, thermocouple position, and LED emission spectrum. Before the reaction, the catalysts were activated in situ in a 10 mL/min flow of gas mixture containing 61% H_2_, 26% CO_2_, and 13% N_2_ at 340 °C for 30 min. Afterward, the flow was adjusted to 50 mL/min. During the light-assisted RWGS tests, the catalyst layer was illuminated by a Schott KL2500 LED source (400 nm < λ < 700 nm), equipped with an optic fiber with a 9 mm active diameter and light-focusing lenses (Thorlabs Inc.), which concentrated the light to a spot equal to the catalyst pellet diameter (4.5 mm) with a maximum intensity of 790 mW/cm^2^, as measured by Thorlabs PM100D photometer. Gas analysis was performed by GC (model 490, equipped with MS5A and PPU columns by Agilent).

## Data Availability

Data will be made available on request.

## Supporting Information Available

CO rate as a function of time for the 4.5CuAl catalyst at 340 °C under dark RWGS conditions; XRD patterns of pure γ-Al_2_O_3_ and as-synthesized Cu/Al_2_O_3_ catalysts; N_2_ adsorption/desorption isotherms and pore size distribution for pure γ-Al_2_O_3_ support and Cu/Al_2_O_3_ catalysts; structural properties of bare γ-Al_2_O_3_ and Cu/Al_2_O_3_ catalysts; Tauc plot analysis of γ-Al_2_O_3_ optical bandgap; 28/44 peak area ratios under light-assisted, thermocatalytic, and blank conditions; difference DRIFT spectra for the 4.5CuAl catalyst; changes of band intensity characteristic of hydroxyl, formate, carbonate surface species and gas phase CO during RWGS reaction over 4.5CuAl catalyst; experimental setup for the in-situ XAS analysis of Cu/γ-Al2O3 catalysts; and detailed information on XAS data acquisition and fitting (PDF)

## References

[ref1] Djinović P.; Schüth F. Energy Carriers Made from Hydrogen. In Electrochemical Energy Storage for Renewable Sources and Grid Balancing; Elsevier, 2015; pp 183–199.

[ref2] Klerk A. d. Fischer–Tropsch Refining; Wiley-VCH Verlag GmbH & Co. KGaA: Weinheim, Germany, 2011.

[ref3] Daza Y. A.; Kuhn J. N. CO2 Conversion by Reverse Water Gas Shift Catalysis: Comparison of Catalysts, Mechanisms and Their Consequences for CO2 Conversion to Liquid Fuels. RSC Adv. 2016, 6 (55), 49675–49691. 10.1039/C6RA05414E.

[ref4] Vovchok D.; Zhang C.; Hwang S.; Jiao L.; Zhang F.; Liu Z.; Senanayake S. D.; Rodriguez J. A. Deciphering Dynamic Structural and Mechanistic Complexity in Cu/CeO2/ZSM-5 Catalysts for the Reverse Water-Gas Shift Reaction. ACS Catal. 2020, 10 (17), 10216–10228. 10.1021/acscatal.0c01584.

[ref5] Su X.; Yang X.; Zhao B.; Huang Y. Designing of Highly Selective and High-Temperature Endurable RWGS Heterogeneous Catalysts: Recent Advances and the Future Directions. J. Energy Chem. 2017, 26 (5), 854–867. 10.1016/j.jechem.2017.07.006.

[ref6] Boll W.; Hochgesand G.; Higman C.; Supp E.; Kalteier P.; Müller W.-D.; Kriebel M.; Schlichting H.; Tanz H. Gas Production, 3. Gas Treating. In Ullmann’s Encyclopedia of Industrial Chemistry; Wiley-VCH Verlag GmbH & Co. KGaA: Weinheim, Germany, 2011.

[ref7] Kopač D.; Likozar B.; Huš M. How Size Matters: Electronic, Cooperative, and Geometric Effect in Perovskite-Supported Copper Catalysts for CO 2 Reduction. ACS Catal. 2020, 10 (7), 4092–4102. 10.1021/acscatal.9b05303. 32953235 PMC7493227

[ref8] Nelson N. C.; Nguyen M. T.; Glezakou V. A.; Rousseau R.; Szanyi J. Carboxyl Intermediate Formation via an in Situ-Generated Metastable Active Site during Water-Gas Shift Catalysis. Nat. Catal. 2019, 2 (10), 916–924. 10.1038/s41929-019-0343-2.

[ref9] Chen C. S.; Cheng W. H.; Lin S. S. Mechanism of CO Formation in Reverse Water-Gas Shift Reaction over Cu/Al2O3 Catalyst. Catal. Lett. 2000, 68 (1–2), 45–48. 10.1023/A:1019071117449.

[ref10] Kunkes E. L.; Studt F.; Abild-Pedersen F.; Schlögl R.; Behrens M. Hydrogenation of CO2 to Methanol and CO on Cu/ZnO/Al2O3: Is There a Common Intermediate or Not?. J. Catal. 2015, 328, 43–48. 10.1016/j.jcat.2014.12.016.

[ref11] Wang X.; Shi H.; Kwak J. H.; Szanyi J. Mechanism of CO2 Hydrogenation on Pd/Al2O3 Catalysts: Kinetics and Transient DRIFTS-MS Studies. ACS Catal. 2015, 5 (11), 6337–6349. 10.1021/acscatal.5b01464.

[ref12] Goguet A.; Meunier F. C.; Tibiletti D.; Breen J. P.; Burch R. Spectrokinetic Investigation of Reverse Water-Gas-Shift Reaction Intermediates over a Pt/CeO2 Catalyst. J. Phys. Chem. B 2004, 108 (52), 20240–20246. 10.1021/jp047242w.

[ref13] Meunier F.; Goguet A.; Hardacre C.; Burch R.; Thompsett D. Quantitative DRIFTS Investigation of Possible Reaction Mechanisms for the Water–Gas Shift Reaction on High-Activity Pt- and Au-Based Catalysts. J. Catal. 2007, 252 (1), 18–22. 10.1016/j.jcat.2007.09.003.

[ref14] Mateo D.; Cerrillo J. L.; Durini S.; Gascon J. Fundamentals and Applications of Photo-Thermal Catalysis. Chem. Soc. Rev. 2021, 50, 2173–2210. 10.1039/D0CS00357C. 33336654

[ref15] Gargiulo J.; Berté R.; Li Y.; Maier S. A.; Cortés E. From Optical to Chemical Hot Spots in Plasmonics. Acc. Chem. Res. 2019, 52 (9), 2525–2535. 10.1021/acs.accounts.9b00234. 31430119

[ref16] Wang Z.; Song H.; Liu H.; Ye J. Coupling of Solar Energy and Thermal Energy for Carbon Dioxide Reduction: Status and Prospects. Angew. Chem., Int. Ed. 2020, 59 (21), 8016–8035. 10.1002/anie.201907443. 31309678

[ref17] Lorber K.; Djinović P. Accelerating Photo-Thermal CO2 Reduction to CO, CH4 or Methanol over Metal/Oxide Semiconductor Catalysts. iScience 2022, 25 (4), 104107 10.1016/j.isci.2022.104107. 35378856 PMC8976152

[ref18] Lorber K.; Zavašnik J.; Sancho-Parramon J.; Bubaš M.; Mazaj M.; Djinović P. On the Mechanism of Visible-Light Accelerated Methane Dry Reforming Reaction over Ni/CeO2–x Catalysts. Appl. Catal., B 2022, 301, 120745 10.1016/j.apcatb.2021.120745.

[ref19] Marimuthu A.; Zhang J.; Linic S. Tuning Selectivity in Propylene Epoxidation by Plasmon Mediated Photo-Switching of Cu Oxidation State. Science 2013, 339, 1590–1593. 10.1126/science.1231631. 23539599

[ref20] Chen G.; Gao R.; Zhao Y.; Li Z.; Waterhouse G. I. N.; Shi R.; Zhao J.; Zhang M.; Shang L.; Sheng G.; Zhang X.; Wen X.; Wu L. Z.; Tung C. H.; Zhang T. Alumina-Supported CoFe Alloy Catalysts Derived from Layered-Double-Hydroxide Nanosheets for Efficient Photothermal CO2 Hydrogenation to Hydrocarbons. Adv. Mater. 2018, 30 (3), 1–8. 10.1002/adma.201704663. 29205526

[ref21] Gong E.; Ali S.; Hiragond C. B.; Kim H. S.; Powar N. S.; Kim D.; Kim H.; In S. I. Solar fuels: research and development strategies to accelerate photocatalytic CO_2_ conversion into hydrocarbon fuels. Energy Environ. Sci. 2022, 15 (3), 880–937. 10.1039/D1EE02714J.

[ref22] Xie B.; Wong R. J.; Tan T. H.; Higham M.; Gibson E. K.; Decarolis D.; Callison J.; Aguey-Zinsou K.-F.; Bowker M.; Catlow C. R. A.; Scott J.; Amal R. Synergistic Ultraviolet and Visible Light Photo-Activation Enables Intensified Low-Temperature Methanol Synthesis over Copper/Zinc Oxide/Alumina. Nat. Commun. 2020, 11 (1), 1615 10.1038/s41467-020-15445-z. 32235859 PMC7109065

[ref23] Wang L.; Ghoussoub M.; Wang H.; Shao Y.; Sun W.; Tountas A. A.; Wood T. E.; Li H.; Loh J. Y. Y.; Dong Y.; Xia M.; Li Y.; Wang S.; Jia J.; Qiu C.; Qian C.; Kherani N. P.; He L.; Zhang X.; Ozin G. A. Photocatalytic Hydrogenation of Carbon Dioxide with High Selectivity to Methanol at Atmospheric Pressure. Joule 2018, 2 (7), 1369–1381. 10.1016/j.joule.2018.03.007.

[ref24] Shoji S.; Peng X.; Yamaguchi A.; Watanabe R.; Fukuhara C.; Cho Y.; Yamamoto T.; Matsumura S.; Yu M.-W.; Ishii S.; Fujita T.; Abe H.; Miyauchi M. Photocatalytic Uphill Conversion of Natural Gas beyond the Limitation of Thermal Reaction Systems. Nat. Catal. 2020, 3 (2), 148–153. 10.1038/s41929-019-0419-z.

[ref25] Cho Y.; Shoji S.; Yamaguchi A.; Hoshina T.; Fujita T.; Abe H.; Miyauchi M. Visible-Light-Driven Dry Reforming of Methane Using a Semiconductor-Supported Catalyst. Chem. Commun. 2020, 56 (33), 4611–4614. 10.1039/D0CC00729C. 32211643

[ref26] Tan T. H.; Xie B.; Ng Y. H.; Abdullah S. F. B.; Tang H. Y. M.; Bedford N.; Taylor R. A.; Aguey-Zinsou K.-F.; Amal R.; Scott J. Unlocking the Potential of the Formate Pathway in the Photo-Assisted Sabatier Reaction. Nat. Catal. 2020, 3 (12), 1034–1043. 10.1038/s41929-020-00544-3.

[ref27] Tahir B.; Tahir M.; Amin N. S. Gold–Indium Modified TiO2 Nanocatalysts for Photocatalytic CO2 Reduction with H2 as Reductant in a Monolith Photoreactor. Appl. Surf. Sci. 2015, 338, 1–14. 10.1016/j.apsusc.2015.02.126.

[ref28] Song C.; Liu X.; Xu M.; Masi D.; Wang Y.; Deng Y.; Zhang M.; Qin X.; Feng K.; Yan J.; Leng J.; Wang Z.; Xu Y.; Yan B.; Jin S.; Xu D.; Yin Z.; Xiao D.; Ma D. Photothermal Conversion of CO 2 with Tunable Selectivity Using Fe-Based Catalysts: From Oxide to Carbide. ACS Catal. 2020, 10 (18), 10364–10374. 10.1021/acscatal.0c02244.

[ref29] Zhao L.; Qi Y.; Song L.; Ning S.; Ouyang S.; Xu H.; Ye J. Solar-Driven Water-Gas Shift Reaction over CuOx/Al2O3 with 1.1% of Light-to-Energy Storage. Angew. Chem., Int. Ed. 2019, 58 (23), 7708–7712. 10.1002/anie.201902324. 30942941

[ref30] Liu F.; Song L.; Ouyang S.; Xu H. Cu-Based Mixed Metal Oxides for an Efficient Photothermal Catalysis of the Water-Gas Shift Reaction. Catal. Sci. Technol. 2019, 9 (9), 2125–2131. 10.1039/C9CY00359B.

[ref31] Aslam U.; Rao V. G.; Chavez S.; Linic S. Catalytic Conversion of Solar to Chemical Energy on Plasmonic Metal Nanostructures. Nat. Catal. 2018, 1 (9), 656–665. 10.1038/s41929-018-0138-x.

[ref32] Zhou L.; Martirez J. M. P.; Finzel J.; Zhang C.; Swearer D. F.; Tian S.; Robatjazi H.; Lou M.; Dong L.; Henderson L.; Christopher P.; Carter E. A.; Nordlander P.; Halas N. J. Light-Driven Methane Dry Reforming with Single Atomic Site Antenna-Reactor Plasmonic Photocatalysts. Nat. Energy 2020, 5 (1), 61–70. 10.1038/s41560-019-0517-9.

[ref33] Fang S.; Hu Y. H. Thermo-Photo Catalysis: A Whole Greater than the Sum of Its Parts. Chem. Soc. Rev. 2022, 51 (9), 3609–3647. 10.1039/D1CS00782C. 35419581

[ref34] Kale M. J.; Avanesian T.; Christopher P. Direct Photocatalysis by Plasmonic Nanostructures. ACS Catal. 2014, 4 (1), 116–128. 10.1021/cs400993w.

[ref35] Govorov A. O.; Richardson H. H. Generating Heat with Metal Nanoparticles. Nano Today 2007, 2 (1), 30–38. 10.1016/S1748-0132(07)70017-8.

[ref36] Kale M. J.; Avanesian T.; Xin H.; Yan J.; Christopher P. Controlling Catalytic Selectivity on Metal Nanoparticles by Direct Photoexcitation of Adsorbate–Metal Bonds. Nano Lett. 2014, 14 (9), 5405–5412. 10.1021/nl502571b. 25111312

[ref37] Lee C.; Park Y.; Park J. Y. Hot Electrons Generated by Intraband and Interband Transition Detected Using a Plasmonic Cu/TiO2 Nanodiode. RSC Adv. 2019, 9 (32), 18371–18376. 10.1039/C9RA02601K. 35515219 PMC9064733

[ref38] Zhou L.; Swearer D. F.; Robatjazi H.; Alabastri A.; Christopher P.; Carter E. A.; Nordlander P.; Halas N. J. Response to Comment on Quantifying Hot Carrier and Thermal Contributions in Plasmonic Photocatalysis. Science 2019, 364, 69–72. 10.1126/science.aaw9545. 31048463

[ref39] Zhao J.; Nguyen S. C.; Ye R.; Ye B.; Weller H.; Somorjai G. A.; Alivisatos A. P.; Toste F. D. A Comparison of Photocatalytic Activities of Gold Nanoparticles Following Plasmonic and Interband Excitation and a Strategy for Harnessing Interband Hot Carriers for Solution Phase Photocatalysis. ACS Cent. Sci. 2017, 3 (5), 482–488. 10.1021/acscentsci.7b00122. 28573211 PMC5445529

[ref40] Zheng B. Y.; Zhao H.; Manjavacas A.; McClain M.; Nordlander P.; Halas N. J. Distinguishing between Plasmon-Induced and Photoexcited Carriers in a Device Geometry. Nat. Commun. 2015, 6 (1), 7797 10.1038/ncomms8797. 26165521 PMC4510964

[ref41] Dow W.-P.; Wang Y.-P.; Huang T.-J. Yttria-Stabilized Zirconia Supported Copper Oxide Catalyst. J. Catal. 1996, 160 (2), 155–170. 10.1006/jcat.1996.0135.

[ref42] Turco M.; Bagnasco G.; Cammarano C.; Senese P.; Costantino U.; Sisani M. Cu/ZnO/Al2O3 Catalysts for Oxidative Steam Reforming of Methanol: The Role of Cu and the Dispersing Oxide Matrix. Appl. Catal., B 2007, 77 (1–2), 46–57. 10.1016/j.apcatb.2007.07.006.

[ref43] Yahiro H.; Nakaya K.; Yamamoto T.; Saiki K.; Yamaura H. Effect of Calcination Temperature on the Catalytic Activity of Copper Supported on γ-Alumina for the Water-Gas-Shift Reaction. Catal. Commun. 2006, 7 (4), 228–231. 10.1016/j.catcom.2005.11.004.

[ref44] James T. E.; Hemmingson S. L.; Ito T.; Campbell C. T. Energetics of Cu Adsorption and Adhesion onto Reduced CeO2 (111) Surfaces by Calorimetry. J. Phys. Chem. C 2015, 119 (30), 17209–17217. 10.1021/acs.jpcc.5b04621.

[ref45] Campbell C. T. The Energetics of Supported Metal Nanoparticles: Relationships to Sintering Rates and Catalytic Activity. Acc. Chem. Res. 2013, 46 (8), 1712–1719. 10.1021/ar3003514. 23607711

[ref46] Liu P.; Wang H.; Li X.; Rui M.; Zeng H. Localized Surface Plasmon Resonance of Cu Nanoparticles by Laser Ablation in Liquid Media. RSC Adv. 2015, 5 (97), 79738–79745. 10.1039/C5RA14933A.

[ref47] Zabilskiy M.; Arčon I.; Djinović P.; Tchernychova E.; Pintar A. In-situ XAS Study of Catalytic N 2 O Decomposition Over CuO/CeO 2 Catalysts. ChemCatChem 2021, 13 (7), 1814–1823. 10.1002/cctc.202001829.

[ref48] Čižmar T.; Lavrenčič Štangar U.; Fanetti M.; Arčon I. Effects of Different Copper Loadings on the Photocatalytic Activity of TiO2-SiO2 Prepared at a Low Temperature for the Oxidation of Organic Pollutants in Water. ChemCatChem 2018, 10 (14), 2982–2993. 10.1002/cctc.201800249.

[ref49] Bobadilla L. F.; Santos J. L.; Ivanova S.; Odriozola J. A.; Urakawa A. Unravelling the Role of Oxygen Vacancies in the Mechanism of the Reverse Water–Gas Shift Reaction by Operando DRIFTS and Ultraviolet–Visible Spectroscopy. ACS Catal. 2018, 8 (8), 7455–7467. 10.1021/acscatal.8b02121.

[ref50] Kantschewa M.; Albano E. V.; Etrtl G.; Knözinger H. Infrared and X-Ray Photoelectron Spectroscopy Study of K2CO3/γ-Al2O3. Appl. Catal. 1983, 8 (1), 71–84. 10.1016/0166-9834(83)80054-2.

[ref51] Yang Y.; Chai Z.; Qin X.; Zhang Z.; Muhetaer A.; Wang C.; Huang H.; Yang C.; Ma D.; Li Q.; Xu D. Light-Induced Redox Looping of a Rhodium/CexWO3 Photocatalyst for Highly Active and Robust Dry Reforming of Methane. Angew. Chem., Int. Ed. 2022, 61 (21), e202200567 10.1002/anie.202200567. 35277912

[ref52] Hu J.; Li Y.; Zhen Y.; Chen M.; Wan H. In Situ FTIR and Ex Situ XPS/HS-LEIS Study of Supported Cu/Al2O3 and Cu/ZnO Catalysts for CO2 Hydrogenation. Chin. J. Catal. 2021, 42 (3), 367–375. 10.1016/S1872-2067(20)63672-5.

[ref53] Lorber K.; Zavašnik J.; Arčon I.; Huš M.; Teržan J.; Likozar B.; Djinović P. CO2 Activation over Nanoshaped CeO2 Decorated with Nickel for Low-Temperature Methane Dry Reforming. ACS Appl. Mater. Interfaces 2022, 14 (28), 31862–31878. 10.1021/acsami.2c05221. 35801412 PMC9305712

[ref54] Onfroy T.; Li W.-C.; Schüth F.; Knözinger H. Surface Chemistry of Carbon-Templated Mesoporous Aluminas. Phys. Chem. Chem. Phys. 2009, 11 (19), 3671 10.1039/b821505g. 19421478

[ref55] De Bellis J.; Ochoa-Hernández C.; Farès C.; Petersen H.; Ternieden J.; Weidenthaler C.; Amrute A. P.; Schüth F. Surface and Bulk Chemistry of Mechanochemically Synthesized Tohdite Nanoparticles. J. Am. Chem. Soc. 2022, 144 (21), 9421–9433. 10.1021/jacs.2c02181. 35604643 PMC9164225

[ref56] Busca G. The Surface of Transitional Aluminas: A Critical Review. Catal. Today 2014, 226, 2–13. 10.1016/j.cattod.2013.08.003.

[ref57] Solymosi F.; Koós A. ´.; Liliom N.; Ugrai I. Production of CO-Free H2 from Formic Acid. A Comparative Study of the Catalytic Behavior of Pt Metals on a Carbon Support. J. Catal. 2011, 279 (1), 213–219. 10.1016/j.jcat.2011.01.023.

[ref58] He N.; Li Z. H. Palladium-Atom Catalyzed Formic Acid Decomposition and the Switch of Reaction Mechanism with Temperature. Phys. Chem. Chem. Phys. 2016, 18 (15), 10005–10017. 10.1039/C6CP00186F. 27005983

[ref59] Acikgoz M.; Harrell J.; Pavanello M. Seeking a Structure–Function Relationship for γ-Al2O3 Surfaces. J. Phys. Chem. C 2018, 122 (44), 25314–25330. 10.1021/acs.jpcc.8b06506.

[ref60] Lam E.; Corral-Pérez J. J.; Larmier K.; Noh G.; Wolf P.; Comas-Vives A.; Urakawa A.; Copéret C. CO2 Hydrogenation on Cu/Al2O3: Role of the Metal/Support Interface in Driving Activity and Selectivity of a Bifunctional Catalyst. Angew. Chem., Int. Ed. 2019, 58 (39), 13989–13996. 10.1002/anie.201908060. 31328855

[ref61] Sun X.-C.; Yuan K.; Hua W.-D.; Gao Z.-R.; Zhang Q.; Yuan C.-Y.; Liu H.-C.; Zhang Y.-W. Weakening the Metal–Support Interactions of M/CeO2 (M = Co, Fe, Ni) Using a NH3 -Treated CeO2 Support for an Enhanced Water–Gas Shift Reaction. ACS Catal. 2022, 12 (19), 11942–11954. 10.1021/acscatal.2c03664.

[ref62] Gokhale A. A.; Dumesic J. A.; Mavrikakis M. On the Mechanism of Low-Temperature Water Gas Shift Reaction on Copper. J. Am. Chem. Soc. 2008, 130 (4), 1402–1414. 10.1021/ja0768237. 18181624

[ref63] Meunier F.; Reid D.; Goguet A.; Shekhtman S.; Hardacre C.; Burch R.; Deng W.; Flytzani Stephanopoulos M. Quantitative Analysis of the Reactivity of Formate Species Seen by DRIFTS over a Au/Ce(La)O2 Water–Gas Shift Catalyst: First Unambiguous Evidence of the Minority Role of Formates as Reaction Intermediates. J. Catal. 2007, 247 (2), 277–287. 10.1016/j.jcat.2007.02.013.

[ref64] Waxenegger J.; Trügler A.; Hohenester U. Plasmonics Simulations with the MNPBEM Toolbox: Consideration of Substrates and Layer Structures. Comput. Phys. Commun. 2015, 193, 138–150. 10.1016/j.cpc.2015.03.023.

[ref65] Johnson P. B.; Christy R. W. Optical Constants of the Noble Metals. Phys. Rev. B 1972, 6 (12), 4370–4379. 10.1103/PhysRevB.6.4370.

[ref66] Ito T.; Yamaguchi H.; Masumi T.; Adachi S. Optical Properties of CuO Studied by Spectroscopic Ellipsometry. J. Phys. Soc. Jpn. 1998, 67 (9), 3304–3309. 10.1143/JPSJ.67.3304.

[ref67] Kresse G.; Hafner J. Ab Initio Molecular Dynamics for Liquid Metals. Phys. Rev. B:Condens. Matter Mater. Phys. 1993, 47 (1), 558–561. 10.1103/PhysRevB.47.558. 10004490

[ref68] Kresse G.; Joubert D. From Ultrasoft Pseudopotentials to the Projector Augmented-Wave Method. Phys. Rev. B:Condens. Matter Mater. Phys. 1999, 59 (3), 1758–1775. 10.1103/PhysRevB.59.1758.

[ref69] Grimme S.; Antony J.; Ehrlich S.; Krieg H. A Consistent and Accurate Ab Initio Parametrization of Density Functional Dispersion Correction (DFT-D) for the 94 Elements H-Pu. J. Chem. Phys. 2010, 132 (15), 154104 10.1063/1.3382344. 20423165

[ref70] Runge E.; Gross E. K. U. Density-Functional Theory for Time-Dependent Systems. Phys. Rev. Lett. 1984, 52 (12), 997–1000. 10.1103/PhysRevLett.52.997.

[ref71] Digne M.; Sautet P.; Raybaud P.; Euzen P.; Toulhoat H. Use of DFT to Achieve a Rational Understanding of Acid/Basic Properties of Gamma-Alumina Surfaces. J. Catal. 2004, 226 (1), 54–68. 10.1016/j.jcat.2004.04.020.

