# Supplementary material for: Light-Assisted Catalysis and the Dynamic Nature of Surface Species in the Reverse Water Gas Shift Reaction over Cu/γ-Al2O3
Source: ACS Appl Mater Interfaces. 2024 Nov 29;16(49):67778–90. doi: 10.1021/acsami.4c15849 (PMC11647765; doi:10.1021/acsami.4c15849)
Supplement: Supplementary file 1 — am4c15849_si_001.pdf [file am4c15849_si_001.pdf]

Supporting Information

Light-assisted catalysis and the dynamic nature of surface species in the reverse water gas shift reaction over Cu/γ-Al_2O_3

Kristijan Lorber[1,2], Iztok Arčon[2,3], Matej Huš[1,5,6], Janez Zavašnik[3], Jordi Sancho-Parramon[4], Anže Prašnikar[1], Blaž Likozar[1], Nataša Novak Tušar[1,2] and Petar Djinović[1,2]*

[1] National Institute of Chemistry, Hajdrihova 19, SI-1000 Ljubljana, Slovenia

[2] University of Nova Gorica, Vipavska 13, SI-5000 Nova Gorica, Slovenia

[3] Jožef Stefan Institute, SI-1000 Ljubljana, Slovenia

[4] Ruđer Bošković Institute, Bijenička cesta 54, 10000 Zagreb, Croatia

[5] Association for Technical Culture of Slovenia, Zaloška 65, SI-1000 Ljubljana, Slovenia

[6] Institute for the Protection of Cultural Heritage, Poljanska 40, SI-1000 Ljubljana, Slovenia

*Corresponding author E-mail: petar.djinovic@ki.si

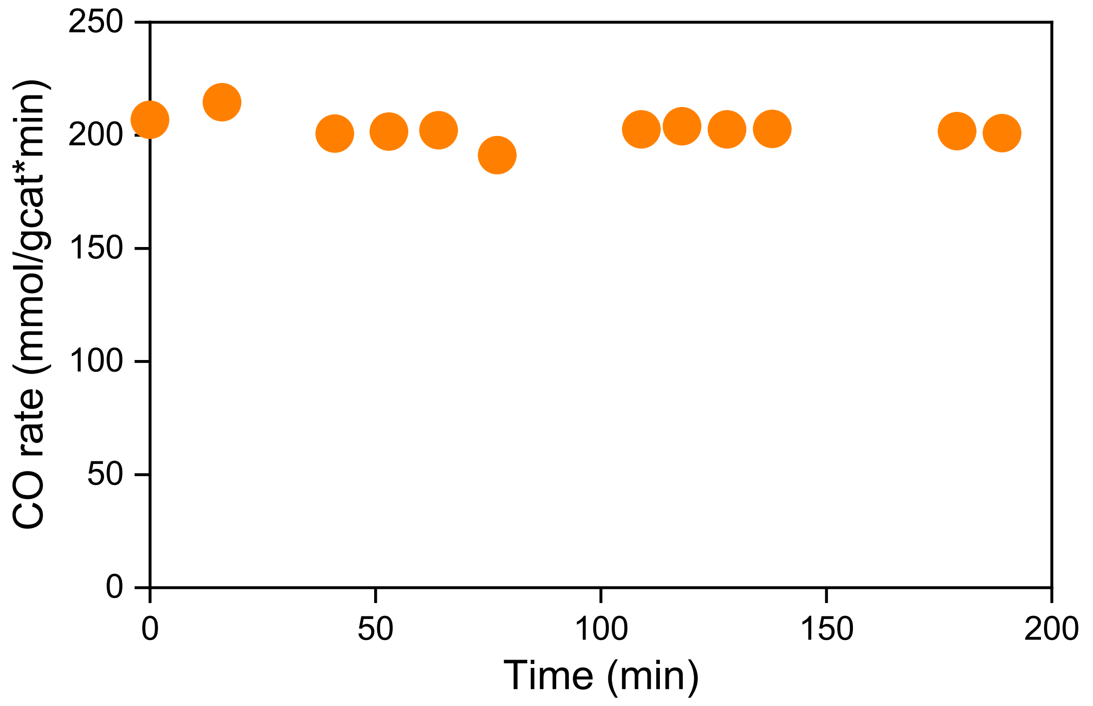

Figure S1. CO rate as a function of time for the 4.5CuAl catalyst at 340 °C under dark RWGS conditions.

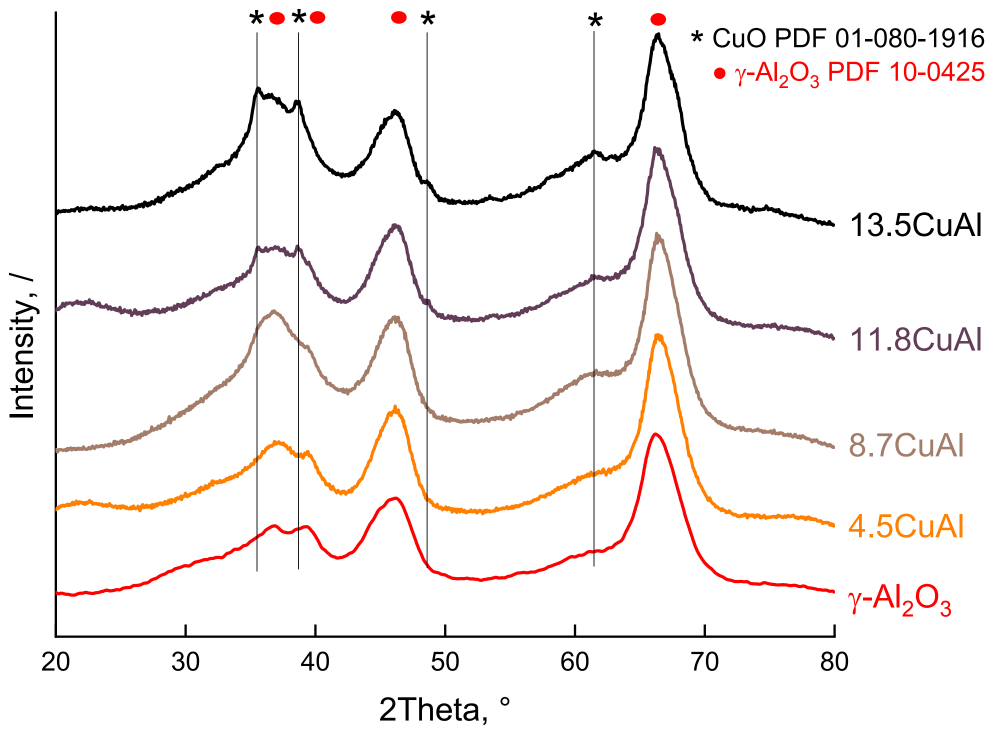

Figure S2. XRD patterns of pure γ-Al_2O_3 and as-synthesized Cu/Al_2O_3 catalysts containing different amounts of copper. Spectra are offset vertically for clarity.

N_2 physisorption

The N_2 sorption analysis for bare γ-Al_2O_3 and Cu/Al_2O_3 catalysts (Figure S3A) yields type IV isotherms, which are characteristic for mesoporous materials. Pore size distribution changed only marginally after copper addition (Figure S3B), whereas pore volume and BET specific surface area decreased progressively with increasing copper content (Table S3).

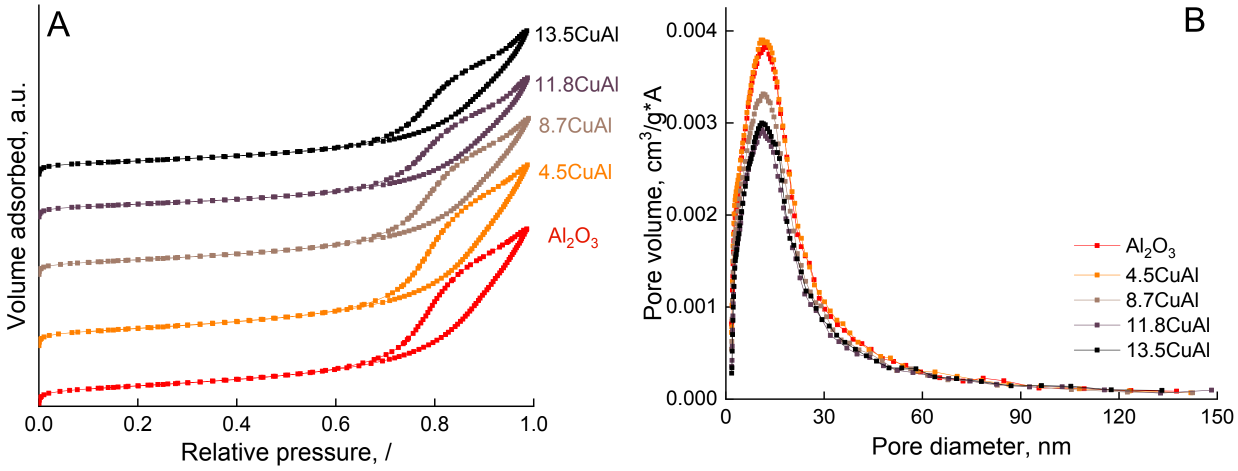

Figure S3. N_2 adsorption/desorption isotherms (a) and pore size distribution for pure γ-Al_2O_3 support and Cu/Al_2O_3 catalysts (b). Isotherms are offset vertically for clarity.

Table S1. Structural properties of bare γ-Al_2O_3 and Cu/Al_2O_3 catalysts.

Sample	BET surface area, m^2/g	Total pore volume, cm^3/g	Average pore size, nm
γ-Al_2O_3	257	0.95	14.7
4.5Cu	262	0.98	14.9
8.7Cu	223	0.85	15.2
11.8Cu	198	0.76	15.2
13.5Cu	202	0.78	15.3

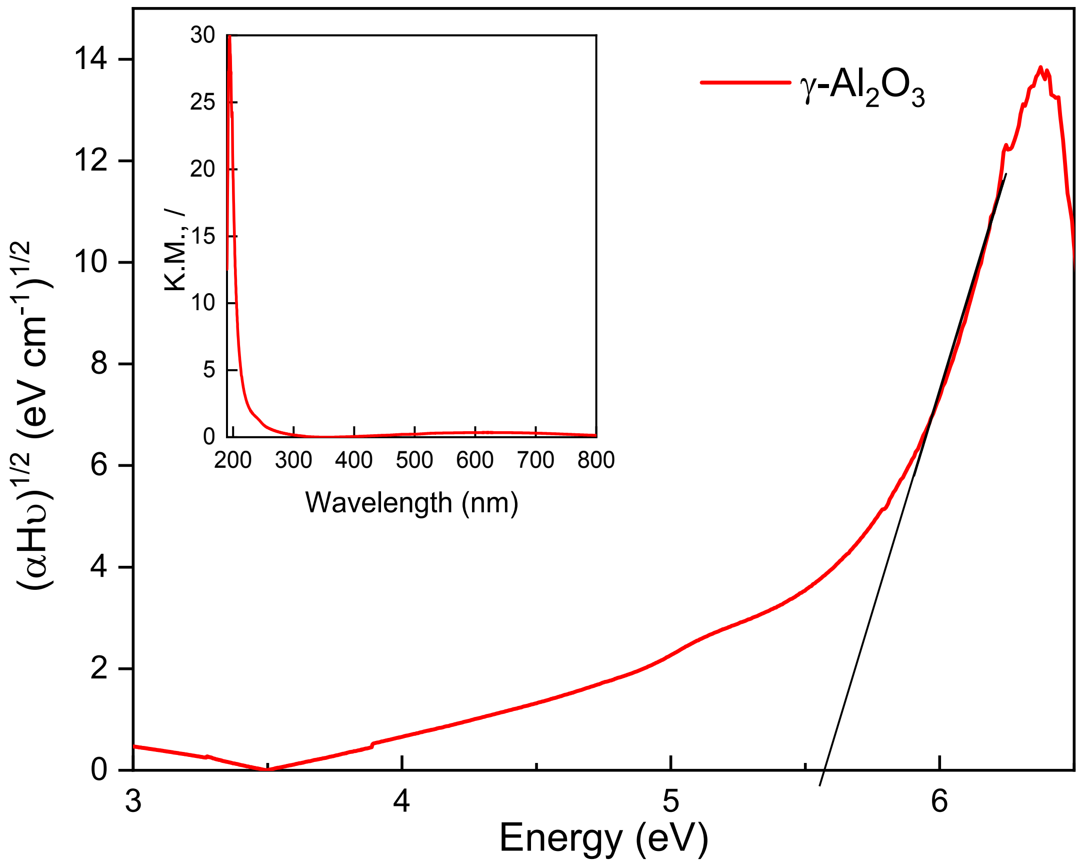

Figure S4. Tauc plot analysis of γ-Al_2O_3 optical bandgap. Inset shows optical absorption.

Electrodynamic properties of Cu/Al_2O_3 materials

Copper particle size effect

We analyzed the scattering cross section, extinction cross section and localized electromagnetic near field enhancement of partially truncated copper particles having different sizes (Figure S5). As the Cu size gradually increases from 5 to 15 and 30 nm, the amount of scattered and absorbed light increases, but the position of the plasmon resonance does not change. For analysis of Cu particles smaller than 5 nm one has to consider the continuous electrodynamic description used here is inappropriate as quantum effects can dominate the optical response.

The electromagnetic near field displays a similar distribution regardless of the Cu size, as it corresponds to the case of particles much smaller than the wavelength (quasi-static approximation). The field distribution simply scales up with the particle size and although the maximum field enhancement is similar in all cases, larger particles provide more extended regions with strong field enhancement.

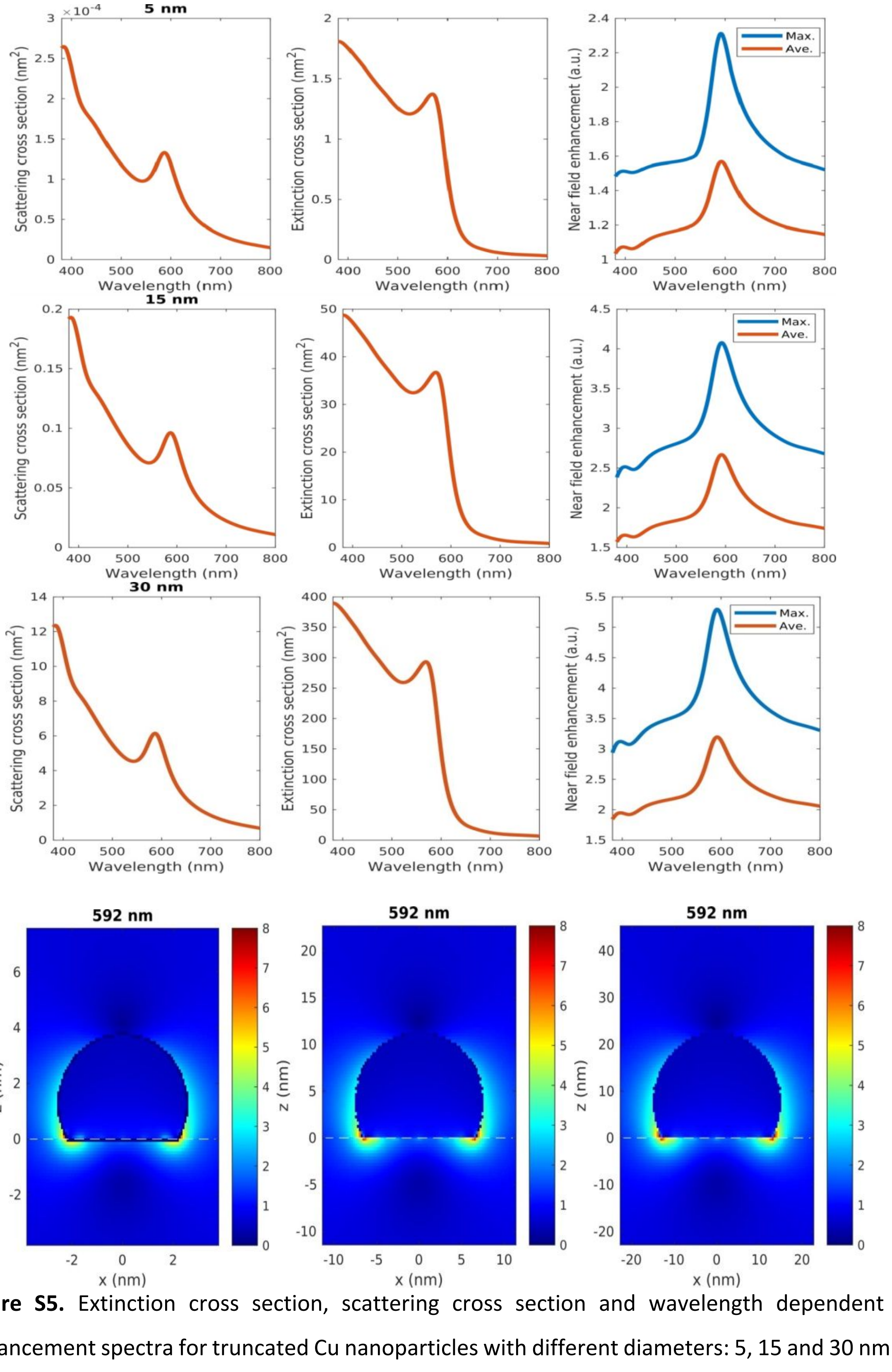

Figure S5. Extinction cross section, scattering cross section and wavelength dependent near field enhancement spectra for truncated Cu nanoparticles with different diameters: 5, 15 and 30 nm. Near field maps (excitation with λ=592 nm) are shown in the bottom panel.

Cu particles on Al_2O_3: truncation effects

When the spherical Cu particle with a 15 nm diameter becomes progressively more truncated (sphere → ¾ sphere → hemisphere), the peak position of the localized plasmon resonance (LSPR) shifts to longer wavelengths (550 nm for a spherical shape and 587 nm for a hemispherical particle), Figure S6. In addition, the plasmon resonance becomes more pronounced with respect to the overall extinction spectra. The electromagnetic near field distribution is also Cu shape dependent (right hand side of Figure S6). Truncation of the Cu particle results in stronger near fields which are more localized at the particle/substrate interface whereas for the spherical Cu particles, the nearfield enhancement mainly covers the nanoparticle surface. Consequently, truncation strengthens both LSPR and electromagnetic nearfield intensity.

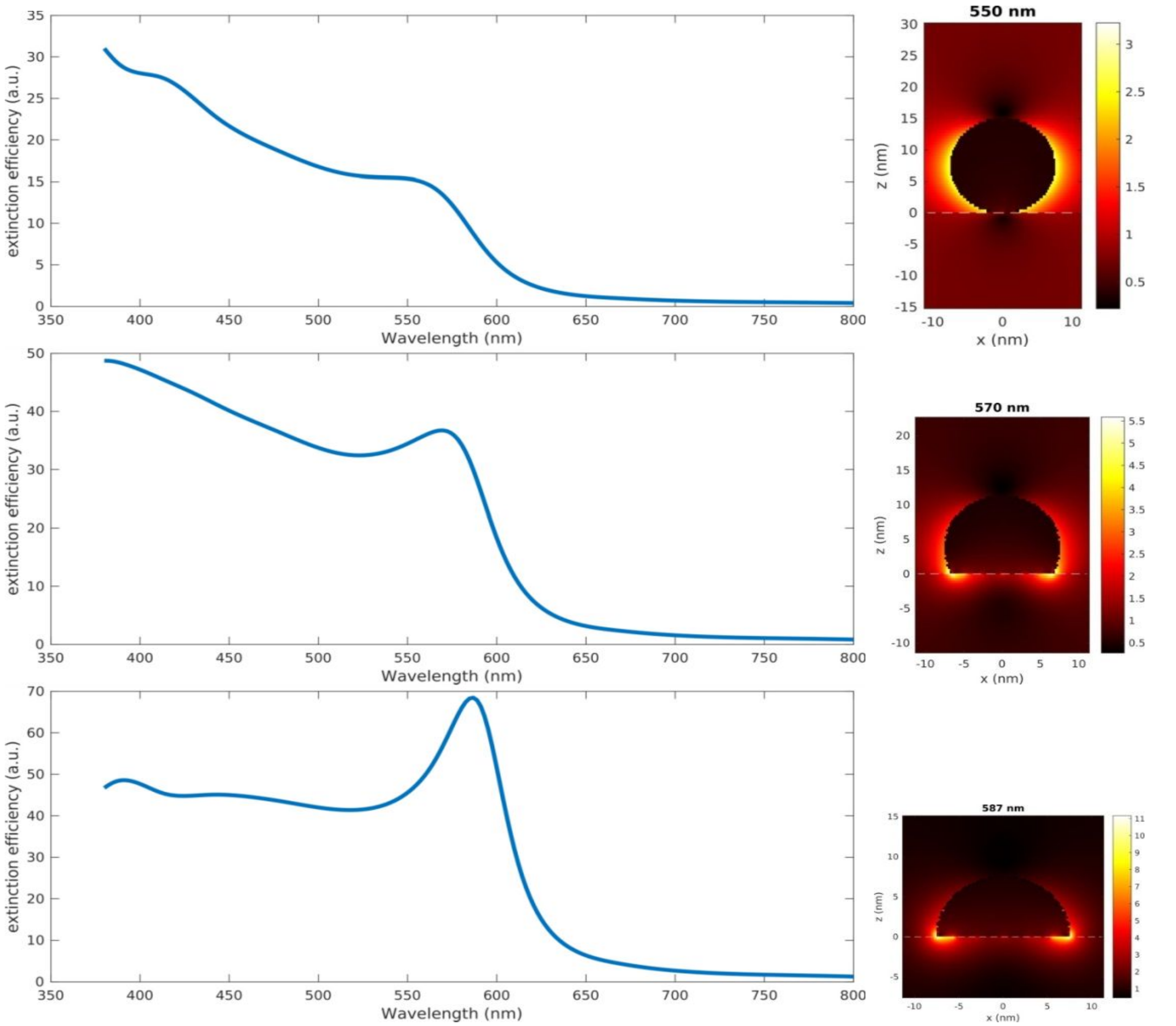

Figure S6. Extinction cross section of Cu nanoparticle (diameter equal to 15 nm) on an Al_2O_3 substrate with different truncations levels: full sphere (top) truncated (middle), hemisphere (bottom).

Pulse experiments to probe the presence of redox RWGS mechanism

Experiments were performed in the Harrick micro-reactor. Prior to analysis, the 4.5CuAl catalyst was *in-situ* reduced in 10 ml/min of 5% H/N_2 flow for 30 min at 340 °C. Then, the flow was switched to argon (30 ml/min) and the sample was heated to 370 °C for 30 min to desorb hydrogen. The sample was cooled back to 340 °C in argon and CO_2 (0.22 ml) was pulsed over the sample using an electronically actuated 6-port valve by Vici. Gas phase (m/z= 44 and m/z=28) was sampled by a mass spectrometer DSMS by Hiden Analytical, UK. Blank experiment was done without the catalyst in dark at 340 °C.

The ratio between integrated m/z=28 and m/z=44 peak areas (Figure S7A) were identical during experiments in dark, under illumination (790 mW/cm^2 of visible light) and blank experiment at 340°C, revealing there is no CO_2 dissociation to CO on 4.5CuAl under tested conditions. Also, CO_2 and H_2 pulse dynamics in dark and under illumination (Figure S7) show no differences in interaction between CO_2 and catalyst surface, whereas H_2 desorbs slightly slower from the illuminated catalyst surface.

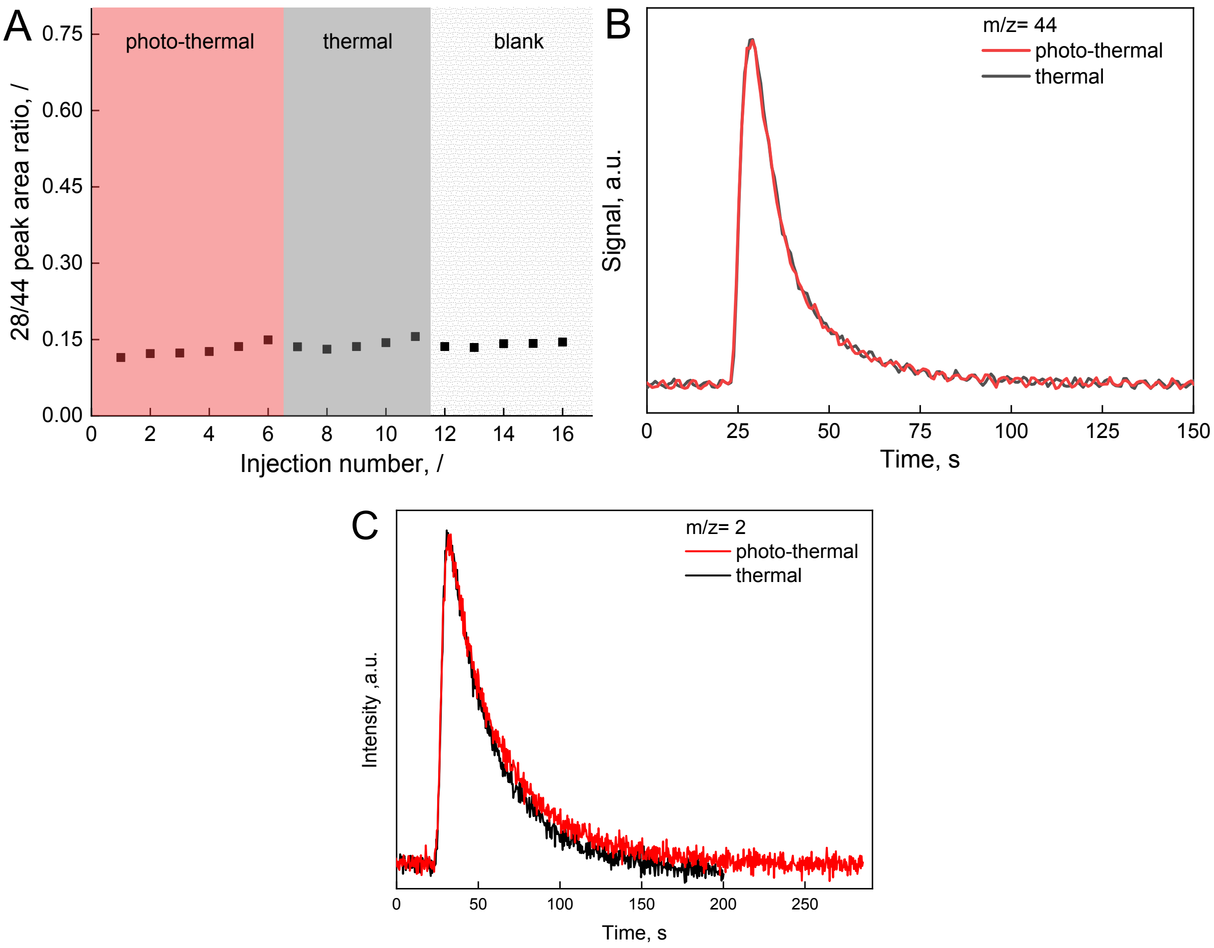

Figure S7. A) The 28/44 peak area ratios under light-assisted, thermocatalytic and blank conditions. B) Pulse CO_2 and C) H_2 dynamics when injected over the activated 4.5CuAl catalyst in argon flow.

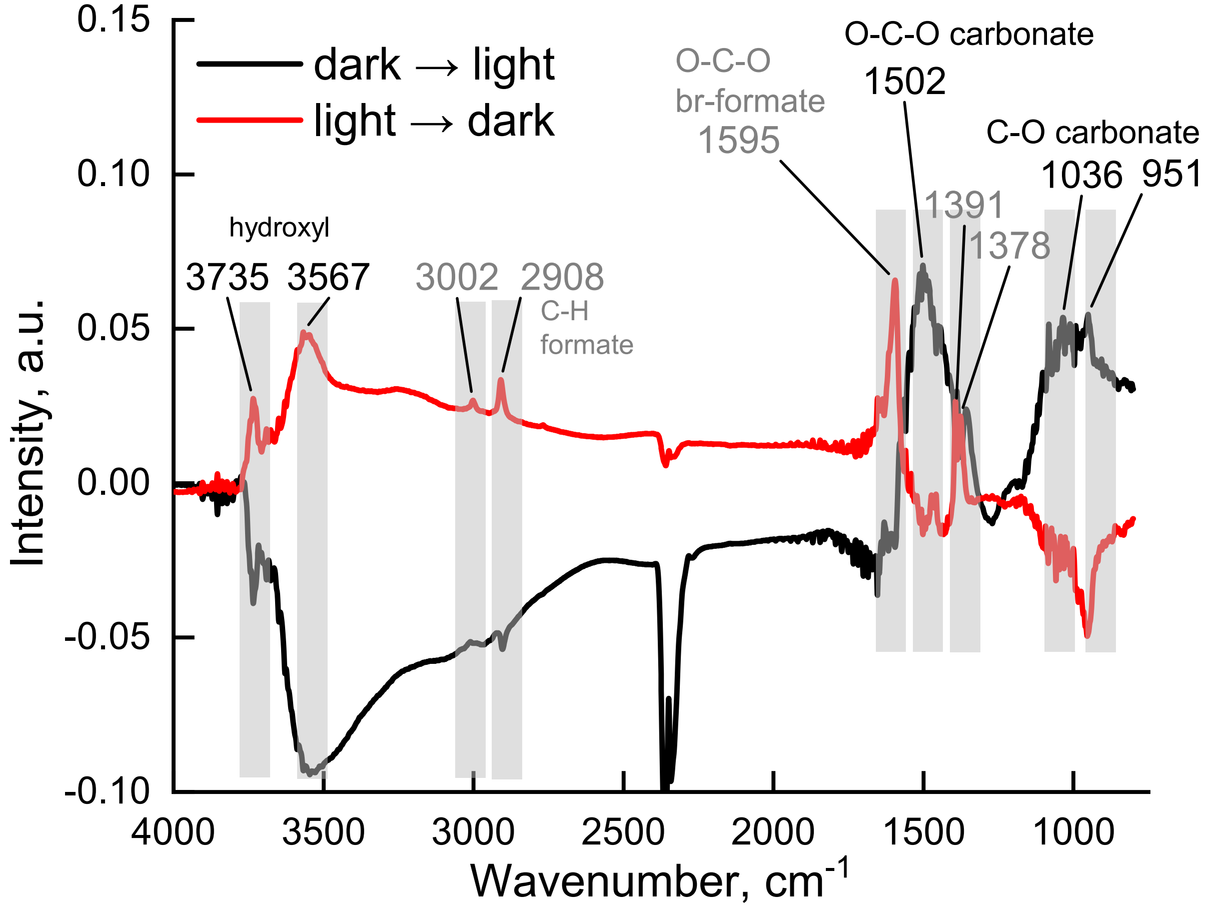

Figure S8. Difference DRIFT spectra for the 4.5CuAl catalyst showing spectral changes occurring during isothermal dark → light (black trace) and light → dark transitions (red trace) at 340 °C. Negative bands represent a lower surface population, positive bands represent a higher surface population.

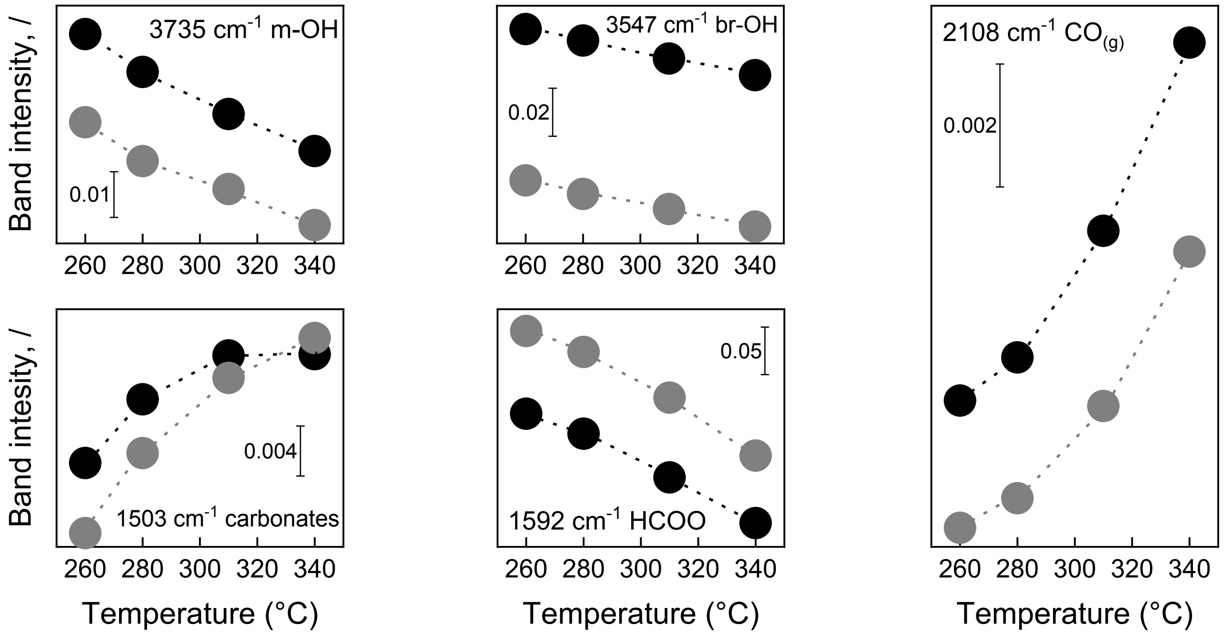

Figure S9. Changes of band intensity characteristic of hydroxyl, formate, carbonate surface species and gas phase CO during RWGS reaction over 4.5CuAl catalyst as a function of catalyst temperature in dark (black symbols) and during light-assisted catalysis (grey symbols). Constant irradiation by 240 mW/cm^2 of white light was used for the light assisted experiment.

***In-situ* Cu K-edge XANES and EXAFS experimental conditions**

Experiments were performed in transmission detection mode at the BM23 beamline of the ESRF synchrotron radiation facility in Grenoble, France. XAS spectra were measured on the as synthesized 4.5CuAl catalyst at RT in helium, after activation in 10% H_2/He stream (flowrate of 30 ml/min) at 1 bar at 340°C, and during the photo-thermal catalytic reaction in CO_2/H_2/He stream (flowrate 7ml/min of H_2, 3ml/min of CO_2 and 20 ml/min of He) at 1 bar at 340°C, with and without visible light illumination of the catalyst. A Si(111) double crystal monochromator was used with energy resolution of about 1 eV at 9 keV. Higher-order harmonics were effectively eliminated by the flat mirror installed in front of the monochromator. The beam size on the sample was 4 mm horizontal and 0.2 mm vertical.

The catalyst samples were prepared in the form of homogeneous micronized powder mixed with BN powder and inserted in the quartz capillary with 1.5 mm diameter. The total X-ray absorption thickness (µd) of about 1.5 was obtained above the investigated Cu K-edge. The quartz capillary with the sample was mounted on the micro-reactor holder (Fig. S1), placed in the monochromatic beam between the first two ionization detectors. XAS spectra were measured on the as synthesized 4.5CuAl catalyst at RT in helium, after activation in 10% H_2/He stream (flowrate of 30 ml/min) at 1 bar at 340°C, and during the photo-thermal catalytic reaction in CO_2/H_2/He stream (flowrate 7ml/min of H_2, 3ml/min of CO_2 and 20 ml/min of He) at 1 bar at 340°C, with and without visible light illumination of the catalyst. The catalyst was heated with hot air and temperature was monitored with thermocouple (K type, O.D.= 0.25 mm) inserted in the quartz capillary. The capillary containing the catalyst powder was irradiated by approximately 400 mW/cm^2 of white light (Schott KL2500 LED, 400 nm $< \lambda <$ 700 nm) emitted from an optic fiber with a 9 mm active diameter, Figure S1.

The Cu K-edge XANES and EXAFS spectra were measured in the energy region from -150 eV to +1000 eV relative to the investigated Cu K-edge. In XANES energy region, 0.3 eV steps were used and equidistant k steps of 0.03 $Å^{-1}$ in EXAFS region, with an integration time of 2 s/step. Two repetitions of each scan were measured and superimposed to improve the signal-to-noise ratio. Exact energy calibration was provided by measurements of absorption spectra on Cu metal foil placed between second and third ionization detectors. The first inflection point in the Cu metal K-edge is set at 8979.0 eV.

The analysis of the EXAFS spectra was performed with the Demeter (IFFEFIT) program package[1] in combination with the FEFF6 program code[2] for ab initio calculation of photoelectron scattering paths. Structural parameters of the average local Cu neighborhood (type and average number of neighbors, the radii and Debye-Waller factor of neighbor shells) are quantitatively resolved from the EXAFS spectra by

comparing the measured EXAFS signal with model signal, constructed *ab initio* with the FEFF6 program code. [2] Combined FEFF models are used, composed of neighbor atoms at distances characteristic for the expected Cu oxide and Cu metal species that may be present in the sample in different catalyst states during the *in-situ* experiment. The atomic species of neighbors are identified in the fit by their specific scattering factor and phase shift.

EXAFS supplementary note

The FEFF model for the initial state of the catalyst comprised oxygen atoms in the nearest coordination shell at two different distances. Al and Cu neighbors are included in more distant coordination shells at distances characteristic for the expected Cu oxide nanoparticles and Cu(II) cations attached to the Al_2O_3 support forming Cu-O-Al bridges. In total six single scattering paths were used (O neighbors at two distances, Al at two distances and Cu at two distances). Three variable parameters are introduced in the model for each scattering path: the coordination number (N), the distance (R) and the Debye-Waller factor (σ2). Debye–Waller factors of the two Al paths and of the two Cu paths were constrained to common values for the same type of atoms. In addition, a common shift of energy origin ΔEo is also allowed to vary. The amplitude-reduction factor S_0^2 is kept fixed at the value of 0.8 in agreement with previous Cu K-edge EXAFS analyses.[3]. A very good agreement between the model and the experimental spectra is found using the k range of 3 –12$Å^{-1}$ and the R range of 1.1 to 4.0 Å. The best fit structural parameters are listed in the Table S1.

The FEFF model for the EXAFS spectra measured *in-situ* during the activation of the catalysts, and during the (photo)catalytic reaction with and without visible light illumination of the catalyst is based on the fcc crystal structure of Cu metal with the lattice constant a = 3.60 Å.[4]. The first three single scattering paths, belonging to the photoelectron scattering on the three nearest Cu coordination shells up to 4.5 Å, are included. In addition, three single scattering paths are added (oxygen neighbors and Al neighbors at two distances), as in the case of the FEFF model for the catalysts measured in the initial state at RT, to test for eventual presence of Cu-O-Al bridges. In total six single scattering paths were used. Three variable parameters are introduced in the model for each scattering path: the coordination number (N), the distance (R) and the Debye-Waller factor (σ2). The Debye–Waller factors of the two Al paths and of the three Cu paths were constrained to common values for the same type of atoms. In addition, a common shift of energy origin ΔEo is also allowed to vary. The amplitude-reduction factor S_0^2 is kept fixed at the value of 0.8. A very good agreement between the model and the experimental spectra is found using the k range of 3 –12$Å^{-1}$ and the R range of 1.1 to 4.6 Å. The best fit structural parameters are listed in Table S2.

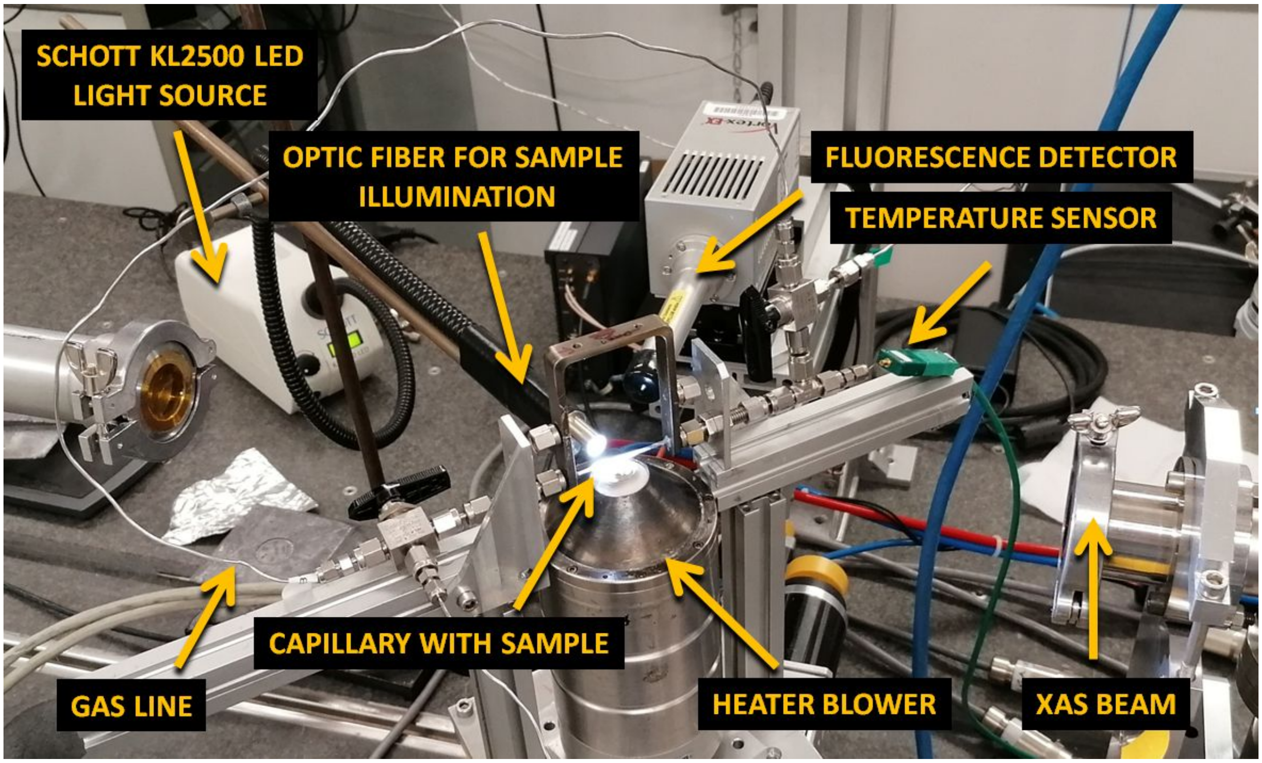

Figure S10. Experimental setup for the *in-situ* XAS analysis of Cu/γ-Al_2O_3 catalysts at ESRF synchrotrone, beamline BM23.

Table S2. Parameters of the nearest coordination shells around Cu cations in the 4.5CuAl catalyst measured in initial state at RT: average number of neighbor atoms (*N*), distance (*R*), and Debye-Waller factor (σ^2). Uncertainty of the last digit is given in parentheses. The best fit is obtained with the amplitude reduction factor S_0^2=0.80 and the shift of the energy origin ΔE_o of 0 eV ±1 eV. *R*-factor (quality of fit parameter) is listed in the last column.

Cu neighbor	*N*	*R* [Å]	σ^2 [Å^2]	ΔE_o/*R*-factor
**4.5CuAl - initial state at RT**				
O	3.5(2)	1.96(6)	0.0056(5)	0.0028
O	2.5(2)	2.28(3)	0.020(5)	
Al	1.0(3)	2.84(2)	0.008(1)	
Al	1.0(3)	3.04(2)	0.008(1)	
Cu	0.6(3)	3.04(2)	0.006(1)	
Cu	0.6(3)	3.57(5)	0.006(1)	

Table S3. Parameters of the nearest coordination shells around Cu cations in the 4.5CuAl catalyst measured after activation in 10% H_2/He stream at 340°C, and during catalytic reaction in CO_2/H_2/He stream

at 340°C with and without visible light illumination of the catalyst: average number of neighbor atoms (*N*), distance (*R*), and Debye-Waller factor (σ^2). Uncertainty of the last digit is given in parentheses. The best fit is obtained with the amplitude reduction factor S_0^2=0.80 and the shift of the energy origin ΔE_o of 0 eV ±1 eV. *R*-factor (quality of fit parameter) is listed in the last column.

Cu neighbor	*N*	*R* [Å]	σ^2 [Å^2]	*R*-factor
**4.5CuAl - after activation in 10% H_2/He stream at 340°C**				
Metallic Cu				
Cu	10(1)	2.51(1)	0.021(1)	0.0065
Cu	5(6)	3.64(2)	0.021(1)	
Cu	5(2)	4.42(2)	0.021(1)	
Cu-O-Al bridges				
O	1.8(4)	2.71(3)	0.004(2)	
Al	1.9(3)	3.66(2)	0.004(1)	
Al	0.9(4)	3.81(2)	0.004(1)	

Cu neighbor	*N*	*R* [Å]	σ^2 [Å^2]	*R*-factor
**4.5CuAl - catalytic reaction in CO_2/H_2/He stream at 340°C – Dark 1**				
Metallic Cu				
Cu	11(1)	2.51(1)	0.021(1)	0.0080
Cu	5(3)	3.71(2)	0.021(1)	
Cu	5(2)	4.45(2)	0.021(1)	
Cu-O-Al bridges				
O	1.8(4)	2.71(3)	0.004(1)	
Al	0.9(6)	3.68(2)	0.003(1)	
Al	2(1)	3.81(2)	0.003(1)	

Cu neighbor	*N*	*R* [Å]	σ^2 [Å^2]	*R*-factor
**4.5CuAl - catalytic reaction in CO_2/H_2/He stream at 340°C - Light**				
Metallic Cu				
Cu	10(1)	2.51(1)	0.020(1)	0.0046
Cu	4(2)	3.65(2)	0.020(1)	
Cu	5(2)	4.41(2)	0.020(1)	
Cu-O-Al bridges				
O	1.5(4)	2.71(3)	0.004(1)	
Al	1.8(6)	3.70(2)	0.004(1)	
Al	0.7(3)	3.87(2)	0.004(1)	

Cu neighbor	*N*	*R* [Å]	σ² [Å²]	*R*-factor
**4.5CuAl - catalytic reaction in CO_2/H_2/He stream at 340°C – Dark 2**				
Metallic Cu				
Cu	10(6)	2.51(1)	0.020(1)	
Cu	5(1)	3.72(2)	0.020(1)	
Cu	5(2)	4.45(2)	0.020 (1)	0.0023
Cu-O-Al bridges				
O	1.4(4)	2.72(3)	0.004(1)	
Al	1.7(6)	3.72(2)	0.004(1)	
Al	1.1(6)	3.86(2)	0.004(1)	

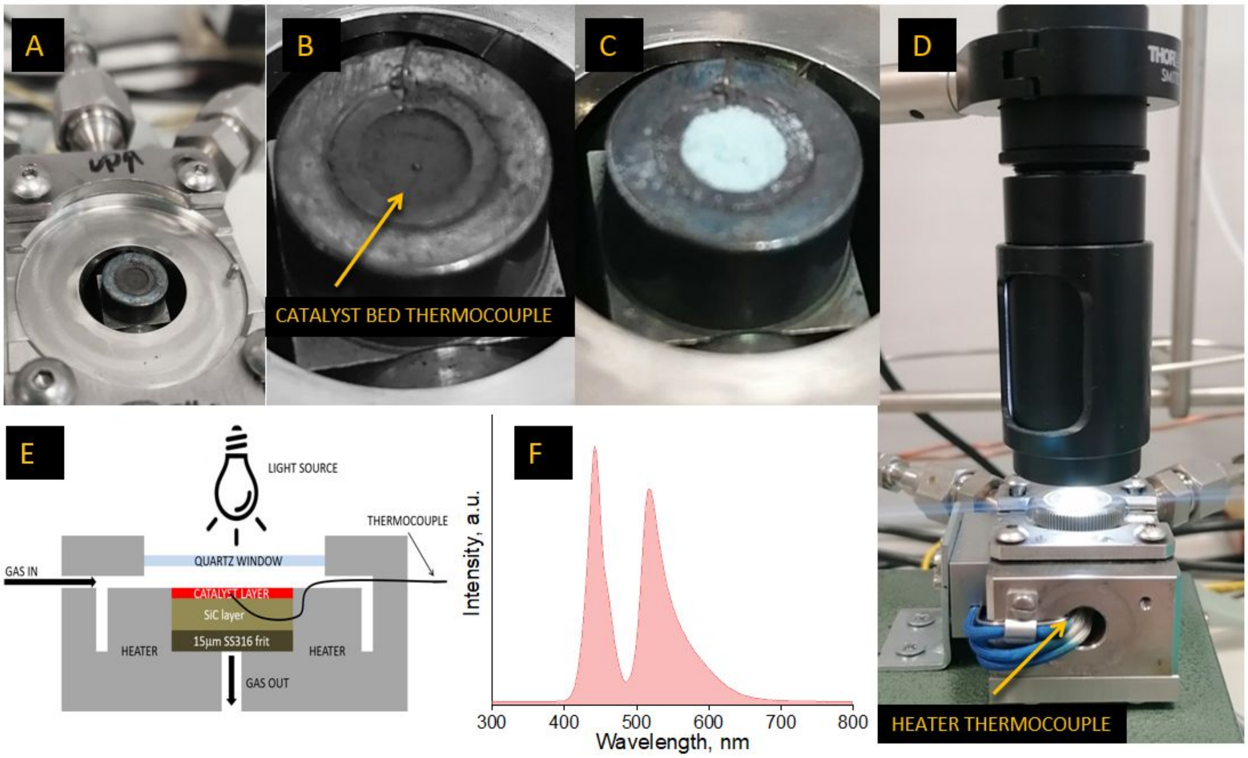

Figure S11. A) Reaction chamber with the SiC layer which was used to support the catalyst bed. B) Magnified heated block with SiC layer showing the protruding thermocouple tip which measures the catalyst bed temperature. C) Powdered catalyst layer inside the reaction chamber. D) Illuminated reaction chamber during light-assisted catalytic reaction. Yellow arrow highlights the position of the second thermocouple which measures the temperature near the heating cartridge. E) Schematic side-view of the reaction chamber showing all crucial elements. F) Emission spectrum of the LED light that was used for all catalytic experiments.

References

1. Ravel, B. & Newville, M. ATHENA , ARTEMIS , HEPHAESTUS : data analysis for X-ray absorption spectroscopy using IFEFFIT. *J. Synchrotron Radiat.* **12**, 537–541 (2005).
2. Rehr, J. J., Albers, R. C. & Zabinsky, S. I. High-order multiple-scattering calculations of x-ray-absorption fine structure. *Phys. Rev. Lett.* **69**, 3397–3400 (1992).
3. Čižmar, T., Lavrenčič Štangar, U., Fanetti, M. & Arčon, I. Effects of Different Copper Loadings on the Photocatalytic Activity of TiO_2-SiO_2 Prepared at a Low Temperature for the Oxidation of Organic Pollutants in Water. *ChemCatChem* **10**, 2982–2993 (2018).
4. Zabilskiy, M., Arčon, I., Djinović, P., Tchernychova, E. & Pintar, A. In-situ XAS Study of Catalytic N_2O Decomposition Over CuO/CeO_2 Catalysts. *ChemCatChem* **13**, 1814–1823 (2021).
